# Supplementary material for: Metal-catalyst-free gas-phase synthesis of long-chain hydrocarbons
Source: Nat Commun. 2021 Oct 12;12:5937. doi: 10.1038/s41467-021-26184-0 (PMC8511129; doi:10.1038/s41467-021-26184-0)
Supplement: Supplementary file 1 — Supplementary Information [file 41467_2021_26184_MOESM1_ESM.pdf]

Supplementary Information

Metal-catalyst-free gas-phase synthesis of long-chain hydrocarbons

Lidia Martínez[1†], Pablo Merino[1†], Gonzalo Santoro[1†], José I. Martínez[1], Stergios Katsanoulis[2], Jesse Ault[3], Álvaro Mayoral[4,5,6], Luis Vázquez[1], Mario Accolla[1,7], Alexandre Dazzi[8], Jeremie Mathurin[8], Ferenc Borondics[9], Enrique Blázquez-Blázquez[10], Nitzan Shauloff[11], Rosa Lebrón-Aguilar[12], Jesús E. Quintanilla-López[12], Raz Jelinek[11], José Cernicharo[13], Howard A. Stone[14], Victor A. de la Peña O'Shea[15], Pedro L. de Andres[1]*, George Haller[2]*, Gary J. Ellis[10]*, José A. Martín-Gago[1,16]*

[1]ESISNA group. Instituto de Ciencia de Materiales de Madrid (ICMM-CSIC), c/ Sor Juana Inés de la Cruz 3, 28049 Madrid, Spain.

[2]Institute for Mechanical Systems, ETH Zurich, Leonhardstrasse 21, 8092 Zurich, Switzerland.

[3]School of Engineering, Brown University, Providence, Rhode Island 02912, USA.

[4]Instituto de Nanociencia y Materiales de Aragon (INMA), Spanish National Research Council (CSIC), University of Zaragoza, 50009 Zaragoza, Spain.

[5]Laboratorio de Microscopias Avanzadas (LMA), University of Zaragoza, 50009 Zaragoza, Spain.

[6]Center for High-Resolution Electron Microscopy (CħEM), School of Physical Science and Technology, ShanghaiTech University, 393 Middle Huaxia Road, Pudong, Shanghai, 201210, China.

[7]Catania Astrophysical Observatory (INAF), Via Santa Sofia, 78, 95123, Catania, Italy.

[8]Institute of Chemical Physics, Université Paris-Saclay, 91400 Orsay, France.

[9]Synchrotron Soleil, L'Orme des Merisiers, Saint-Aubin - BP 48, 91192 Gif-sur-Yvette, France.

[10]Instituto de Ciencia y Tecnología de Polímeros, ICTP-CSIC, c/ Juan de la Cierva 3, 28006 Madrid, Spain.

[11]Department of Chemistry, Ben Gurion University of the Negev, Beer Sheva 84105, Israel

[12]Instituto de Química-Física Rocasolano (IQFR-CSIC), c/ Serrano 119, 28006 Madrid, Spain.

[13]Instituto de Física Fundamental (IFF-CSIC), c/ Serrano 123, 28006 Madrid, Spain.

[14]Department of Mechanical and Aerospace Engineering, Princeton University, Princeton, New Jersey 08544, USA.

[15]Photoactivated Processes Unit IMDEA Energía, Av. Ramón de la Sagra, 3 28935 Móstoles, Spain.

[16]Institute of Physics of the CAS, Cukrovarnicka 10, Prague, Czech Republic.

This PDF file includes:

Supplementary Notes (Note 1 - Note14)
Supplementary Figures 1 to 24
Supplementary Table 1)
Supplementary References 1 to 43

Table of Contents

Supplementary Notes

1.- Experimental chambers and reaction heads

2.- Transition from nanoparticles to a full mat of fibres. Efficiency and scaling considerations

3.- Evidence that fibres do not grow via surface diffusion

4.- Temperature considerations

5.- XPS characterization

6.- Micro-Raman characterization

7.- AFM-IR characterization

8.- Supplementary discussion on fibres and hydrocarbon chains

9.- Ab-initio Density Functional Theory: details and Formation and stability of CH_2

10.- Molecular dynamics: details and the reaction $C + H_2 \rightarrow CH_2$

11.- Methylene Polymerization

12.- Carbon nanoparticle considerations and modelling

13.- Alkane detachment mechanism

14.- Reconstruction of the flow geometry in the magnetron head

Supplementary Note 1: Experimental chambers and reaction heads

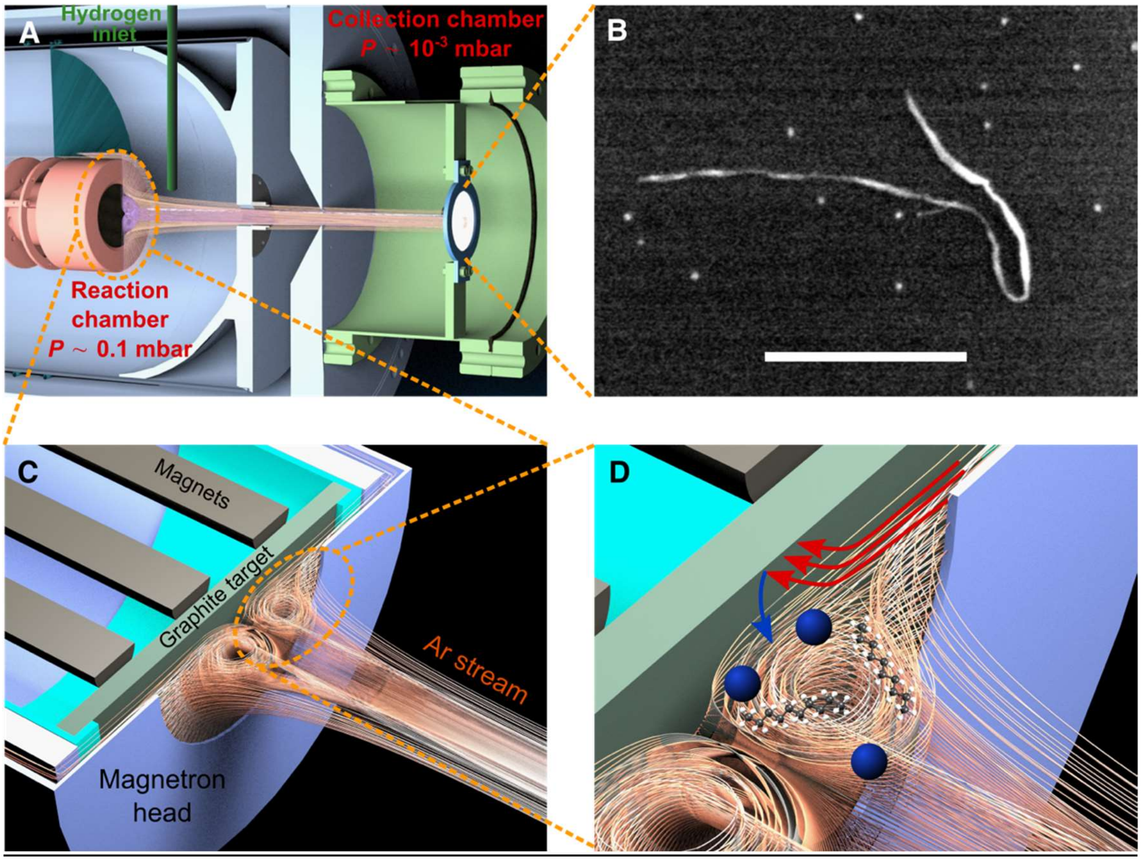

Supplementary Figure 1. General schematic of the gas aggregation source and overall process: (A) The vacuum chamber is comprised of two independent chambers: *Reaction chamber*, where hydrocarbons are formed and Collection chamber, where the fibres are collected on a surface and analysed *in situ* or removed for *ex situ* analysis. The Ar flow, used for the sputtering process, drags the formed material from the reaction chamber to the collection, where it can be either analysed *in situ* or extracted once collected on a surface for *ex situ* analysis. The distance from the target to the collection point is 80 cm. Using this technology, we vaporize C atoms from a graphite target in a gas-phase reaction chamber, an aggregation zone with special inlets that allows the direct introduction of gases, such as H_2.[1] (B) *ex situ* SEM image of a typical individual fibre collected on a silicon Si(100) surface with its native oxide (scalebar 500 nm). The bright spots correspond to amorphous C-nanoparticles. (C) Zoom of the magnetron-head, with orange lines indicating Ar atom trajectories. A three-dimensional fluid-flow structure creates a trap-and-release mechanism that forces the ejected atoms to increase their residence time while protected by the mean Ar stream. (D) Further zoom of the sputter magnetron head: detail of the Ar^+ ions impact (red arrows) towards the graphite target and the possible trajectory of knock out C atoms ejected towards the attraction areas (blue arrow), where gas phase C–C coupling occurs to form C-nanoparticles (blue spheres) and alkane chains (not at scale).

Supplementary Note 2: Transition from nanoparticles to a full mat of fibres. Efficiency and scaling considerations

Previous experiments using this experimental set-up [2] concluded that graphite sputtering leads to the aggregation of C atoms generating amorphous C-nanoparticles with an average size distribution of 9.3 nm and a standard deviation of 0.3 nm (Supplementary Figure 2A), as well as hydrocarbons formed with the residual hydrogen of the vacuum system. In ref. [3] some TEM images showing the amorphous nature of the material are presented. However, when H_2 is introduced into the reactor at higher densities than that of the ejected C atoms, the structure of the products obtained changes radically. Long fibres, of up to 2.5 microns in length ranging from 6 to 20 nm in diameter, are formed (Figs. S1B and S2B-C). Supplementary Figure 2C shows a spherical aberration corrected (Cs-corrected) high-resolution transmission electron microscope (HR-TEM) image of a single fibre collected on a holey carbon TEM grid that rules out the possibility that its growth occurs on a surface, and Supplementary Figure 2D, depicted a detail of the individual fibre of Fig. 1D, where it can be observed that in the fibres coexist amorphous regions with those that are highly crystalline. In the crystalline regions, chains up to 36 atoms were found (red rectangle Supplementary Figure 2D) reaching up to ≈ 8.2 nm; additionally, although it is not very common longer chains, especially on the surface of the fibres can be also observed, demonstrating that it is comprised of highly entangled short chains.

Moreover, the fibres collected on a silicon oxide surface form a mat of overlapping fibres that can be seen in places to agglomerate and fuse together. We estimate from the image in Fig S2E, and from other AFM images, a lower limit for the density of 10^{10} fibres/cm^2. From this value, we can estimate a lower total deposition rate of 10^8 fibres/minute cm^2 at a distance of 80 cm from the target. Additionally, in this respect it should be considered that the value is also dependent on the nature of the collection surface. For instance, images recorded in Supplementary Figure 3 were all obtained simultaneously, and the adhesion is clearly higher on the graphite surface.

The figure of merit used in Fischer-Tropsch synthesis is the average conversion, which is typically of 45-50% reaching in some particular cases 90% [4] and the control of selectivity to long chain hydrocarbons, which predicted by the ASF law cannot exceed 45%.[5] In our case, although difficult to estimate, we have a conversion higher than 45% and a selectivity as high as 70% for C24 +, values comparable to those obtained by Fischer-Tropsch synthesis. Moreover, although efficiency in terms of energy introduced in the system is also very difficult to assess, some hints can be also given. Our magnetron requires a power of about 75W, and the process takes place in a low-pressure environment (therefore, low gas consumption). Laboratory based Fischer-Tropsch experiments require electrical power to maintain the temperature of the system above 500K (which could also be around 200W) and a higher consumption of the reagents (high pressure). Also, remembering that "conventional" industrial FTS converts “syngas” (a mixture of carbon monoxide and hydrogen obtained from the gasification of natural gas, coal or biomass) to alkanes, then the efficiency is also highly dependent on the feedstock. Gasification is the most costly and inherently inefficient and energy demanding process (typically >1000 K), and the efficiency of the feedstock is biomass < coal < natural gas <<< petroleum refining.

All these considerations and numbers suggest that after further optimization of the experimental and geometrical parameters of the system, the process could be upscaled for technological production. Especially interesting points to consider for scaling are:

- The easy recovery and reuse of argon and unreacted hydrogen gas
- The recovery of C-NPs for use in other applications
- No need for costly purification methods or the elimination of additives or other chemicals

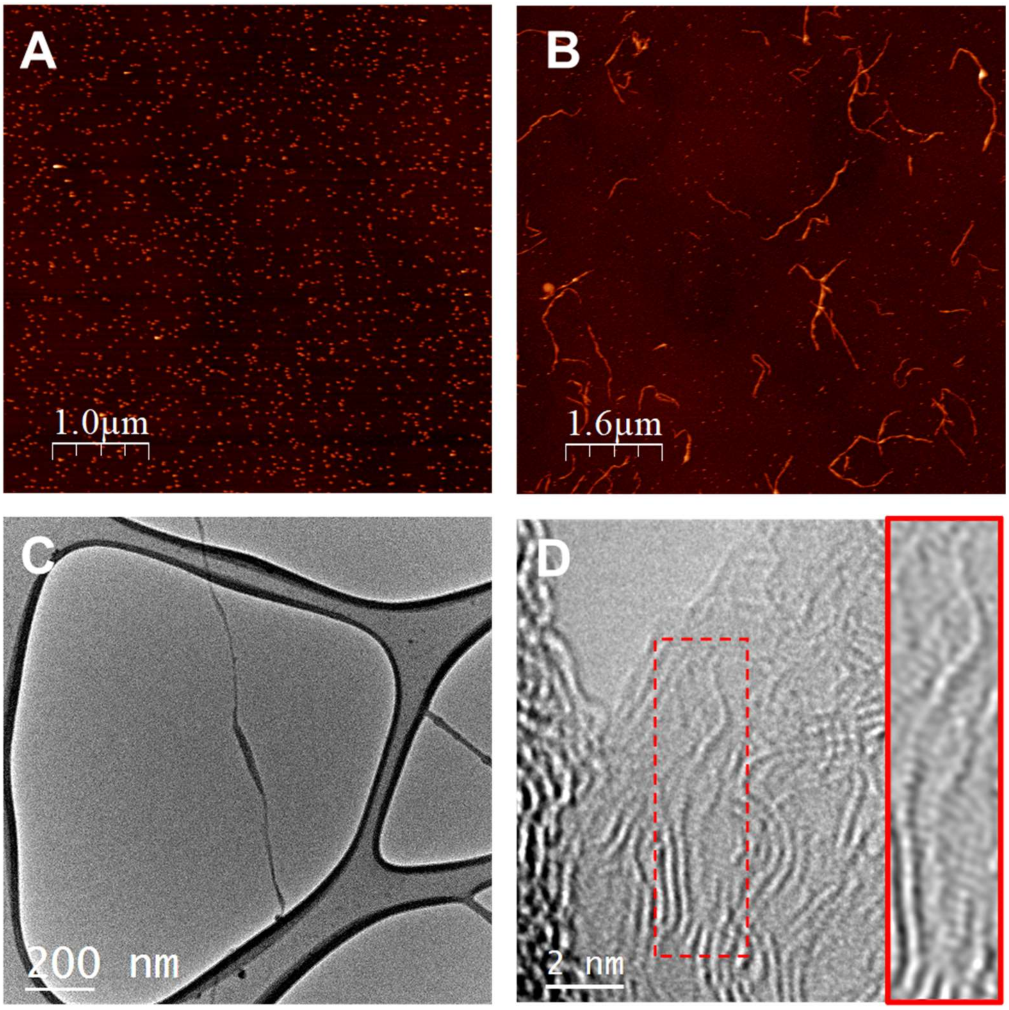

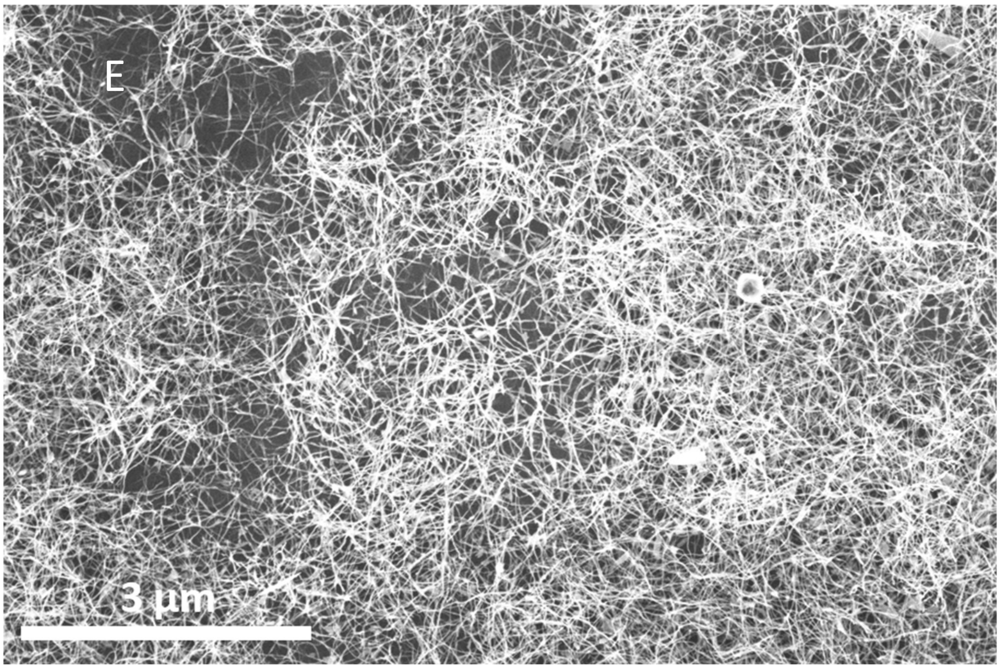

Supplementary Figure 2. Structural characterization of the fibres. (A) AFM image of C nanoparticles obtained without H_2 injection, in contrast with (B) fibres observed when H_2 is injected. (C) Low magnification TEM image of a fibre suspended on a holey carbon grid. (D) C_s-corrected HR-TEM image corresponding to a detail of the fibre of Fig. 1C, where the locally ordered regions can be distinguished. A detailed inspection of this image reveals long carbon chains of over 30 atoms, as highlighted with the example in the red rectangle. (E) mat of fibres collected over 100 minutes on a silicon oxide surface

Supplementary Note 3: Evidence that fibres do not grow via surface diffusion

This section is devoted to demonstrate, firstly, that the fibres do not form, neither on the substrate surface nor on the walls of the UHV chamber. Although the main evidence comes from the fact the fibres form *without* the implication of a surface (see Supplementary Figure 2C), we have performed experiments to clearly show that the surface composition or roughness do not play any important role.

Supplementary Figure 3 presents a series of fibres collected on different substrates, in particular, on a Si wafer with its native oxide, polycrystalline Au and Highly Oriented Pyrolytic Graphite (HOPG). The samples were fabricated using the same experimental conditions. It can be observed clearly that the fibres are present on all substrates, irrespective of the nature of the substrate. For instance, in the case of polycrystalline Au, the fibres are significantly longer than the grain size; see also Supplementary Figure 2C, where a captured fibre is supported on a holey carbon TEM grid.

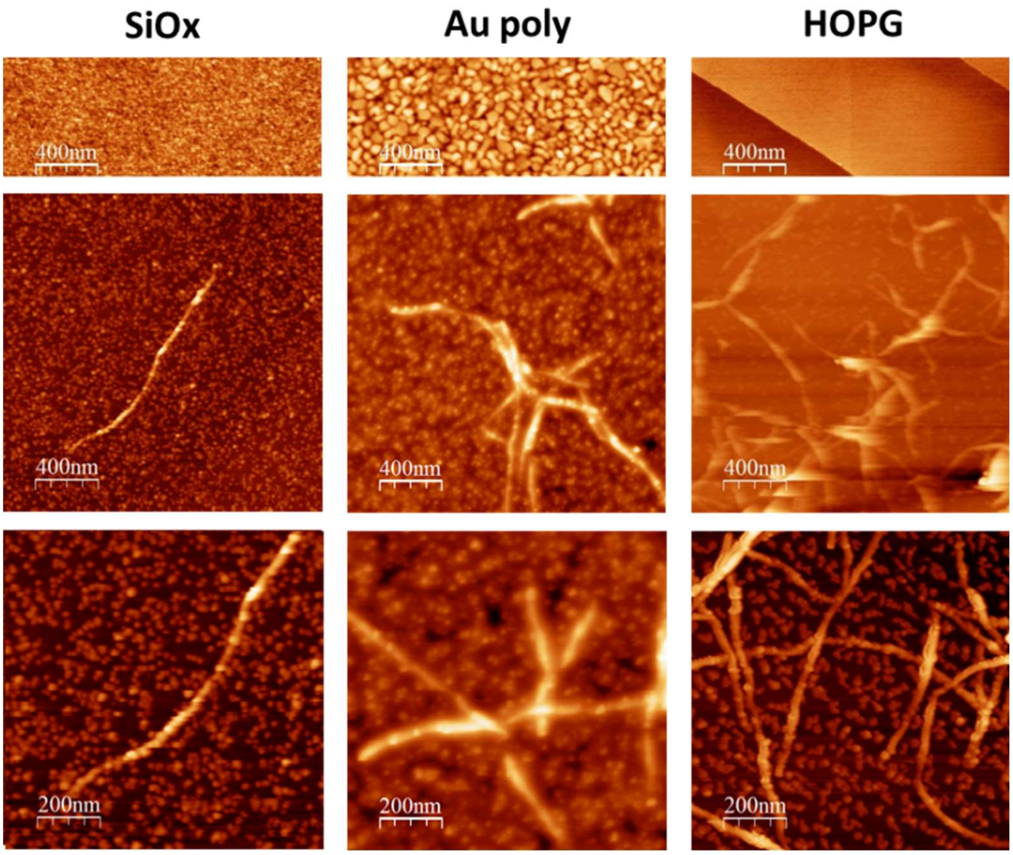

Supplementary Figure 3. AFM images of (top) three different substrates and the resulting deposits of C-structures synthesized injecting 0.15 sccm of H_2 in the Reaction chamber at (middle) lower and (bottom) higher magnification.

Additionally, another fact that provides evidence that the growth does not take place on the sample surface is the observation of overlapping fibres like those observed in Supplementary Figure 3. Moreover, due to their length if they were formed on the walls, they would remain stuck to the wall surfaces.

Also, it can be shown that the interaction of the substrate with the fibres is weak, since both the nanoparticles (NP) and the fibres, can be easily swept away when scanning with the AFM in contact mode (Supplementary Figure 4).

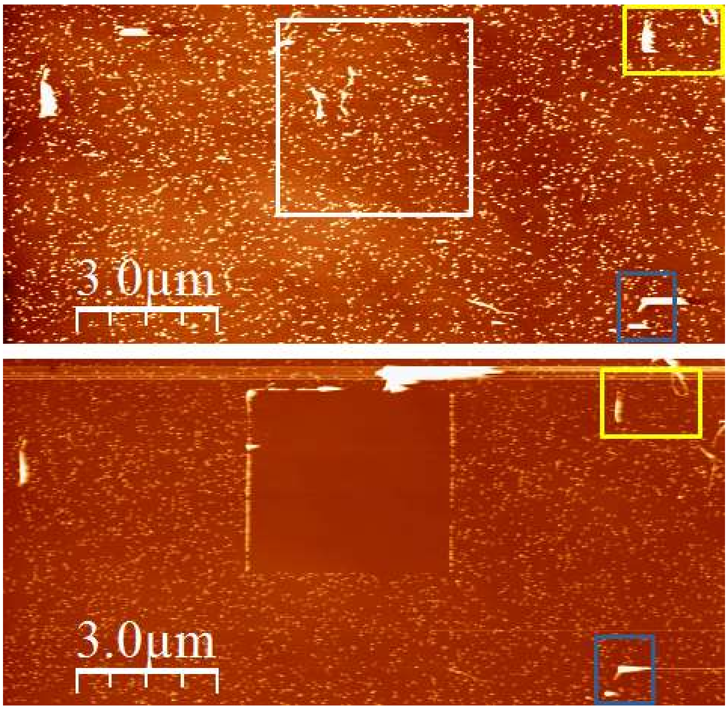

Supplementary Figure 4. AFM image of the carbonaceous deposit on SiO_x where the square marked in white on the top image has been swept by measuring in contact mode. The resulting image is shown in the bottom image. The rectangles in blue and yellow indicate representative features on the surface to identify the region measured.

Although gas aggregation sources have been used mainly with metals, it is a known fact that only a small fraction of sputtered atoms leaves the aggregation zone, as part of them are either redeposited on the cathode, or attached to the cooled chamber walls of the aggregation region, which become coated. [6,7] In our case, a pre-sputtering process is carried out before every run to fully cover the walls and, thus, ensure the same experimental conditions.

Supplementary Note 4: Temperature considerations

The actual temperature during growth in a sputtering gas aggregation sources system at each stage is still an open issue. The magnetron head receives about 100W that are dissipated through a cooling network. The region confined close to the magnetron comprises a cold-plasma, i.e. both the ion and neutral species in the plasma have energies corresponding to about room temperature or some hundred K above. On the other hand, the main jet of Ar is expanding at room temperature and it will thermalize the sputtered material, so it is reasonable to consider that the temperature of the material sputtered from the target will be at maximum the same as that of the gas in the magnetron plasma. [8]

In plasma physics the rotational temperature of the system can be assimilated to the kinetic energy of the gas, since temperature relaxation via collision is a very effective process. In our set-up, we have previously performed [9] optical emission spectroscopy of the magnetron plasma at a distance of 10 mm from the target surface (note that the saddle point is formed at 6.3 mm) using the same conditions as those used in this work, i.e. similar power and Ar flow rate, same injection geometry of H_2 and same H_2 flow rates. From a Boltzmann plot analysis of the population of Q-branch (Δ J=0) rotational levels of H_2,[10] we have determined the average rotational temperature of the plasma to be about 450 K, which could be assimilated to a maximum value of the kinetic energy of the gas.

This value, 450K, although higher than room temperature, is lower than, for example, those typically employed in typical Fischer-Tropsch synthesis protocols, where the structure of the catalyst, the reagents and the selectivity of the reaction strongly determine the used temperature. For instance, in typical reactors, temperatures of about 500K are considered as low temperature Fischer-Tropsch processes and at about 600K high temperature Fischer-Tropsch. [11,12]

Supplementary Note 5: XPS characterization

XPS spectra of the C-structures (nanoparticles and fibres) are measured after depositing them on an atomically clean Au(111) substrate. The experiments were performed in-situ in the XPS end station of the Collection chamber, which ensured the cleanness of the substrate and absence of ambient contamination. In Supplementary Figure 5 we present an overview of the nanoparticle (black) and fibre (red) systems together with a reference spectrum of a clean Au(111) substrate (yellow) shown for reference. The spectra reveal the high purity of the carbon products and the absence of any traces of other chemical elements in the products. Only C 1*s* intensity appears upon deposition and we note the absence of any metal trace.

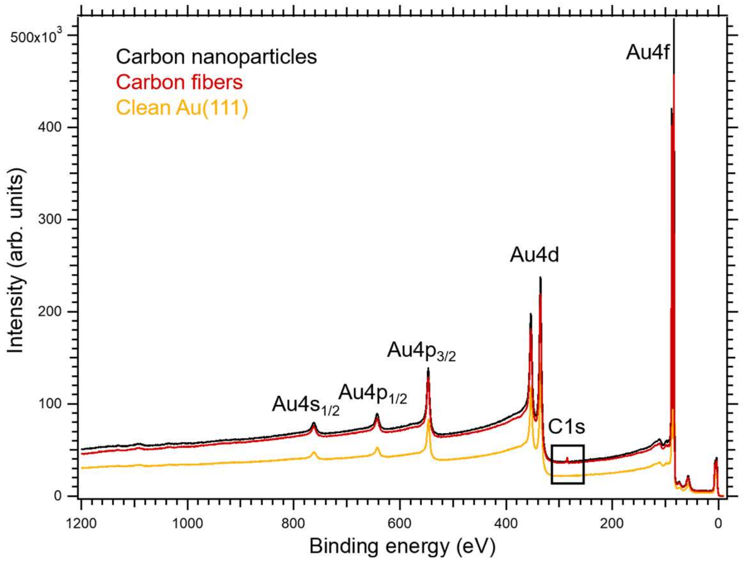

Supplementary Figure 5. XPS survey spectra of the products for without H_2 injection (black) and after H_2 injection (red) hydrogen concentrations during magnetron sputtering. The spectrum of the clean Au(111) surface is shown for comparison (yellow). Only peaks related to Au and C are present in the spectra demonstrating the absence of any metal contamination on the products.

In Supplementary Figure 6 we show a detail of the C 1*s* region. The spectra have been normalized to account for the different deposition time. Thus, the difference in the intensity displayed on the fibres with respect the spectrum obtained on the nanoparticles is an indication of the high efficiency of alkane formation when higher doses of hydrogen are present during the growth of the products in the Reaction chamber.

Importantly, the C 1*s* peaks shown in Supplementary Figure 6 show no signals related to carbon bonded to metals. Metal carbides are reported in the literature to appear in the energy range between 280.8 eV and 283 eV. [13] The region where high intensity is expected is marked in orange. As no intensity occurs, we conclude that our fibres are solely formed of C and H atoms and that no external contamination, which may serve as a traditional catalyst is present even at the level of traces, was found.

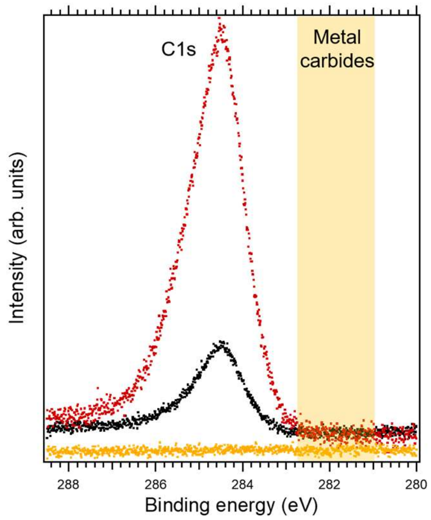

Supplementary Figure 6 High resolution XPS spectra of the C 1*s* region. The spectra have been normalized to account for the different time deposition of the products (100 min for the nanoparticles and 60 min for the fibres). The spectrum of the clean Au(111) surface is shown for comparison (yellow).The C 1*s* peak related to the paraffin fibres (red) present higher intensity, which is consistent with the higher efficiency to form the fibres in a high hydrogen concentration environment and are compared with the case where only residual H_2 gas is present in the UHV Reaction chamber during growth (black). The two situations are analogous to the ones shown in AFM images in Supplementary Figure 2A and Supplementary Figure 2B.

Supplementary Note 6: Micro-Raman characterization.

For the Raman characterization, we collected samples from the Reaction chamber, for which a specific sample transfer system was designed ad-hoc. This allowed comparison between the material synthesized in the Reaction chamber and the C-structures obtained in the Collection chamber. As is described in detail below, the very high chemical similarity between the samples from the Reaction and Collection chambers provide definitive evidence that the alkanes are formed in the Reaction chamber.

Figure S7 presents a representative series of Raman spectra obtained from different samples and recorded at different sample points, compared with the spectra obtained from several solid-state crystalline samples of standard *n*-alkanes, which were selected based on the results obtained from GC-MS (see Fig. 2C in main text).

As can be seen from Supplementary Figure 7 and the corresponding Raman peak assignment in Supplementary Table 1, the similarity in the spectra is quite remarkable, albeit there are several details that require deeper comment.

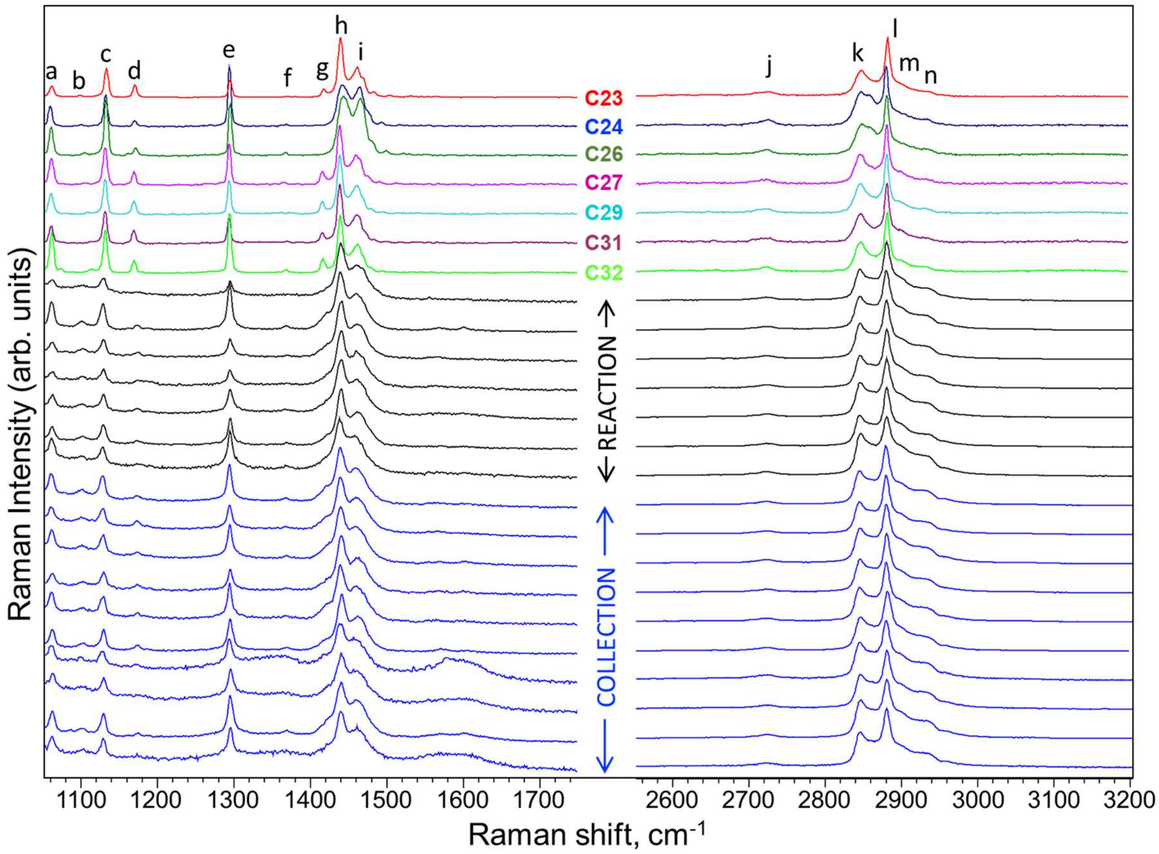

Supplementary Figure 7. Direct comparison between the Raman spectra of several *n*-alkanes with a representative series of spectra obtained from samples deposited on a SiO_x substrate directly inside the Reaction chamber (black spectra) and the Collection chamber (blue spectra) of our experimental set-up, as indicated in Supplementary Figure 1. The bands marked with the letters **a** to **n** correspond to the most characteristic vibrational modes of alkane chains, which are described in Supplementary Table 1.

Supplementary Table 1. Average Raman peak frequencies obtained from curve deconvolution for standard *n*-alkanes (only odd have been considered) and samples grown in our experimental set-up along with their known band assignments.

Peak ID (Fig.S5)	Average band position for the odd *n*-alkanes (cm^{-1})	Average band position, our samples (cm^{-1})	Band assignments [14–18]
a	1061	1062	ν_{as}(C-C)
b	1107	1101	ν (C-C)
c	1132	1128	ν_s(C-C)
d	1170	1174	ρ (CH_2)
e	1295	1295	τ (CH_2)
f	1370	1369	ω (CH_2)
g	1417	1422	ω (CH_2) *F, ov*
h	1439	1440	δ (CH_2) *F, ov*
i	1462	1461	δ (CH_2) *F, ov*
j	2723	2721	ω (CH_2) *ov*
k	2847	2846	ν_s(CH_2)
	2860	2861	ν_s(CH_2) *F*
l	2882	2881	ν_{as}(CH_2) *F*
m	2900	2896	ν_s(CH_3) *F*
	2921	2914	ν_s(CH_2) *F*
n	2934	2934	ν_{as}(CH_3) *F*

Key to vibrational assignments: $\nu_{s,as}$ = symmetric, asymmetric stretching, δ = bending, ρ = rocking, τ = twisting, ω = wagging, *ov* = overtone band, *F* = Fermi resonance.

The bands observed in the spectra obtained from our samples closely correspond with those of the standard *n*-alkane samples, and their assignment to well-accepted vibrational modes in long chain alkanes and polyethylene [14–18] is evident. In particular, the bands at 1062 cm^{-1} (a) and 1130 cm^{-1} (c) are assigned to the symmetric and asymmetric C-C skeletal stretching modes, respectively, the 1295 cm^{-1} peak (e) corresponds to the CH_2 twisting mode, whilst the bands at 1440 cm^{-1} (h) and 1461 cm^{-1} (i) correspond to CH_2 deformation modes. It is well-known that in the CH stretching region the assignments are highly complicated due to significant band overlapping of overtones along with Fermi resonances, [19] however, the main peaks at 2846 cm^{-1} (k) and 2881 cm^{-1} (l) correspond to CH_2 symmetric and asymmetric stretching modes, respectively, and the bands at 2896 cm^{-1} (m) and 2934 cm^{-1} (n) can be assigned to the CH_3

symmetric and asymmetric stretching modes. The band at 2721 cm^{-1} (j) corresponds to the first overtone of the CH_2 wagging mode. [15]

It is important to point out that the *n*-alkane Raman spectra presented here were recorded from a series of highly crystalline solids that over the range between C_{23} – C_{32} present characteristic bands that correspond, in most cases, to an orthorhombic crystalline phase, with some exceptions since the even-number *n*-alkanes (up to C_{28}) have a monoclinic crystalline structure. Thus, due to a different disposition of the alkane chains in the unit cell, the Raman spectra differ, [14] with the particularly notable absence of the band (marked g) at around 1418 cm^{-1} and some slight band frequency shifts in other modes, as can be seen for the spectra of C_{24} and C_{26} in Supplementary Figure 7. Nevertheless, a close correspondence exists between the spectra obtained in our experimental set-up and the standard *n*-alkane spectra. Indeed, the high frequency region shown between 2600 cm^{-1} and 3200 cm^{-1} is almost identical in all cases, and all of the characteristic bands indicated can be clearly identified in the spectra of our samples. However, some differences are apparent that are discussed briefly below.

Firstly, and probably most visible is the lower intensity of the band at 1418 cm^{-1} (marked g). Since the material formed in our experiment is presumably composed of a blend of alkanes of different chain lengths according to the GC-MS results, a sum of the spectra from the different crystalline structures (orthorhombic and monoclinic) would certainly lead to a relatively lower intensity of this band. However, it is far more likely that the band-shape in this region is due to the significantly lower crystallinity of the material produced. The origin of the 1418 cm^{-1} is due to Fermi resonance between overtone and combination bands of the correlation split methylene rocking modes, [14,15,17] giving rise to the characteristic Raman active Ag mode that only appears in the Raman spectra when two adjacent methylene chains in an all-trans conformation lie in the unit cell structure of an orthorhombic crystal. The relative intensity of this band (g) has been traditionally used to measure the crystallinity in long chain alkanes and polyethylene, [20] but in an amorphous material, such as that observed in the melt phase, this band is completely absent. This phenomenon is illustrated in Figure S8, where the Raman spectra of heptacosane ($C_{27}H_{56}$) recorded in the semicrystalline solid-state and in the melt, using a Linkam THMS600 heating stage in combination with a long focal length 50× (NA=0.50) objective, are compared with two spectra recorded on representative samples from the Reaction and Collection chambers. Thus, considering the broadening of the 1295 cm^{-1} band (e) due to the increased contribution of the amorphous band that appears at 1305 cm^{-1} [18,20] and the general broadening and lower definition in the 1418 cm^{-1} region of our grown samples, the spectra clearly support the semicrystalline aliphatic nature of the fibres that were measured from samples obtained both from the Reaction and Collection chambers. This is also in agreement with the appearance of large areas of entangled alkane chains in the HR-TEM data (Figures 2 d,e and Supplementary Figure 2D) together with the ordered regions that correspond to small crystallites.

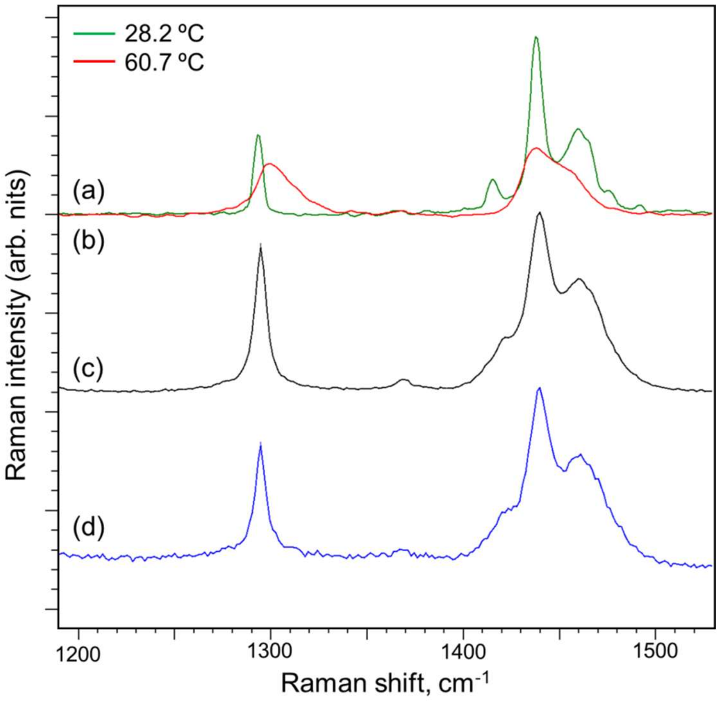

Supplementary Figure 8. Raman spectra of heptacosane ($C_{27}H_{56}$) in (a) crystalline solid state (green) and (b) melt phase (red) compared with samples from our experimental set-up obtained from (c) the Reaction chamber and (d) the Collection chamber.

Finally, in many spectra obtained from our experimental set-up in the range from 1500 cm^{-1} to 1700 cm^{-1}, a broad feature centred around 1590 cm^{-1} can be observed. Selected spectra more clearly illustrating this are provided in Supplementary Figure 9, where a broad feature can also be appreciated near 1360 cm^{-1}. These broad bands can be ascribed to the so-called G and D bands of amorphous carbon, [21] which appear in some spectra due to the presence of the C-nanoparticles that are also formed in our experiment. Statistically the appearance of these bands appears to be slightly higher in the spectra of samples obtained from the Collection chamber.

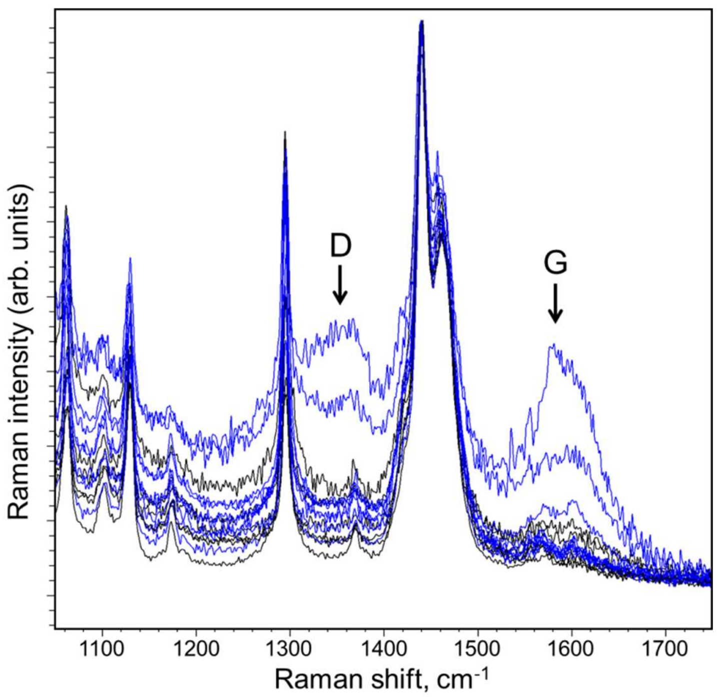

Supplementary Figure 9. Raman spectra illustrating the presence of amorphous carbon in some of the samples obtained from the Reaction chamber (black spectra) and the Collection chamber (blue).

Supplementary Note 7: AFM-IR characterization

AFM-IR is a versatile hybrid technique first developed by Dazzi et al. [22] that combines a pulsed IR laser source with a fully-functional AFM enabling the chemical characterization of materials with monolayer sensitivity and a lateral resolution better than 10 nm. [23–25] The infrared spectrum is generated from the measurement of the photothermal-induced resonance (PTIR) of the cantilever close to the sample that occurs at the vibrational frequencies of the sample, and whose amplitude is proportional to the absorbance.[22,24] AFM-IR in tapping mode allows not only high spatial resolution spectral characterization at specific locations but soft materials can be imaged. Using a Au-coated cantilever in combination with a Au substrate, the enhancement effect induced between the tip and the gold substrate [26] was combined with heterodyne AFM where multiple flexural eigenmodes of the AFM cantilever are measured to enhance the sensitivity of the probe–sample interaction.[27] By observing the cantilever ringdown at each point, the AFM image and the absorption signal at a selected wavenumber can be obtained simultaneously, and by scanning the tip a map of the chemical feature of interest is obtained that directly correlates with the morphological information from the AFM.

Supplementary Figure 10 shows an example of AFM topography obtained from a fibre-rich area on a sample collected on a polycrystalline Au substrate in the Collection chamber.

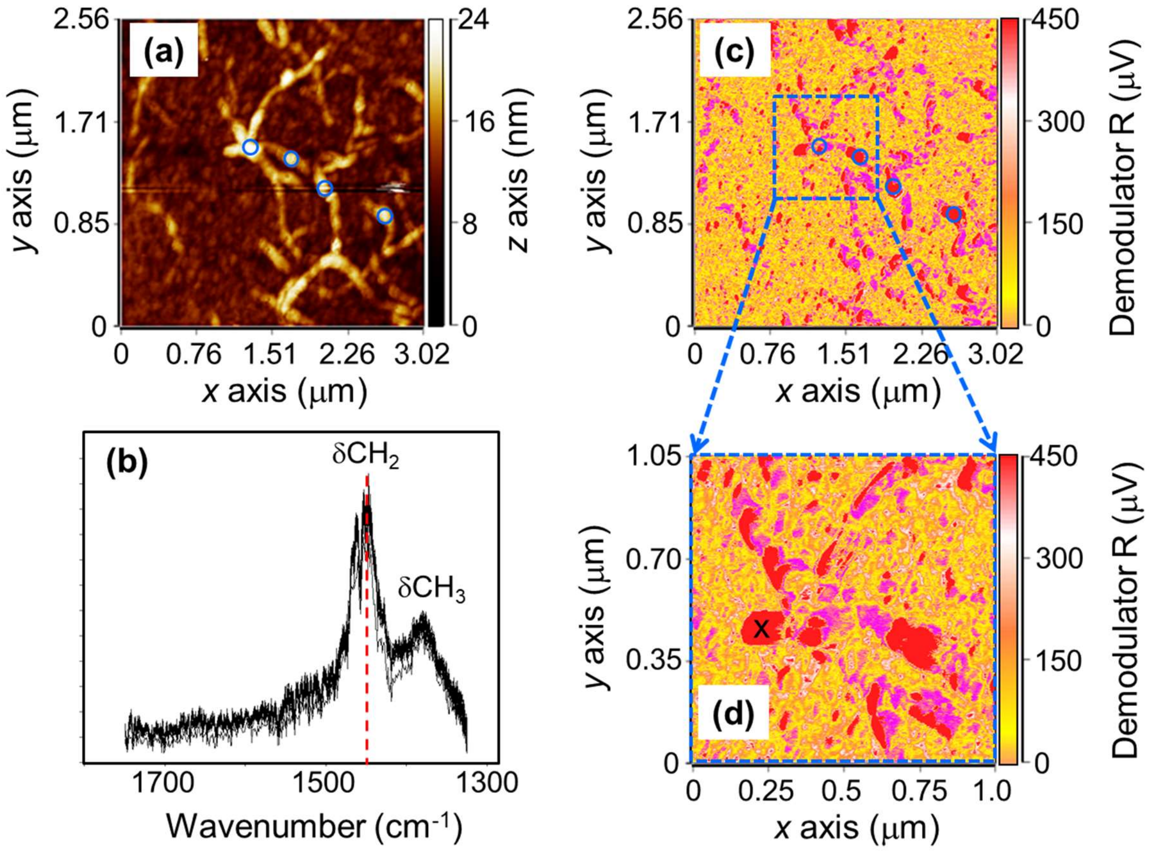

Supplementary Figure 10. AFM-IR study of fibres produced with a H_2 flow rate of 0.15 sccm and deposited on a polycrystalline Au substrate in the Collection chamber. (a) Topography AFM image, (b) spectra recorded from several positions (marked with blue circles) on the samples,

(c) single frequency image corresponding to the intensity of the CH_2 deformation at 1450 cm^{-1} (shown by red dotted line in (b)), and (d) zoom of the area indicated by the blue box in (c).

The morphology of the fibres measured on this AFM, as deduced from the AFM topographic image in Supplementary Figure 10a, was found to be almost identical to that observed from standard AFM images (see main text Fig. 1B and Supplementary Figure 2B) with similar average height values and lateral dimensions, suggesting fibre diameters in the order of 10 – 20 nm. The spectra in Supplementary Figure 10b were obtained from different positions marked by blue circles on the AFM image, and show characteristic methylene and methyl deformation modes (see below for further discussion). Using the demodulated difference signal and scanning the tip at a laser wavelength corresponding to the maximum intensity of the methylene deformations at 1450 cm^{-1}, the IR absorption maps in Supplementary Figures 8c and 8d were generated. These correlate closely to the AFM morphological data of the fibres, with a higher intensity where the fibres overlap and the thickness (height) more than doubles.

In order to analyse the spectral data, the spectrum of Fig. 2e in the main text, corresponding to the position marked "x" in Supplementary Figure 10d, is compared in Supplementary Figure 11 with several conventional FTIR spectra from standard *n*-alkanes. It is worth noticing that the CH_2 signal out of the fibres, corresponding to the background of Supplementary Figures 10c and 10d, is very low and constant.

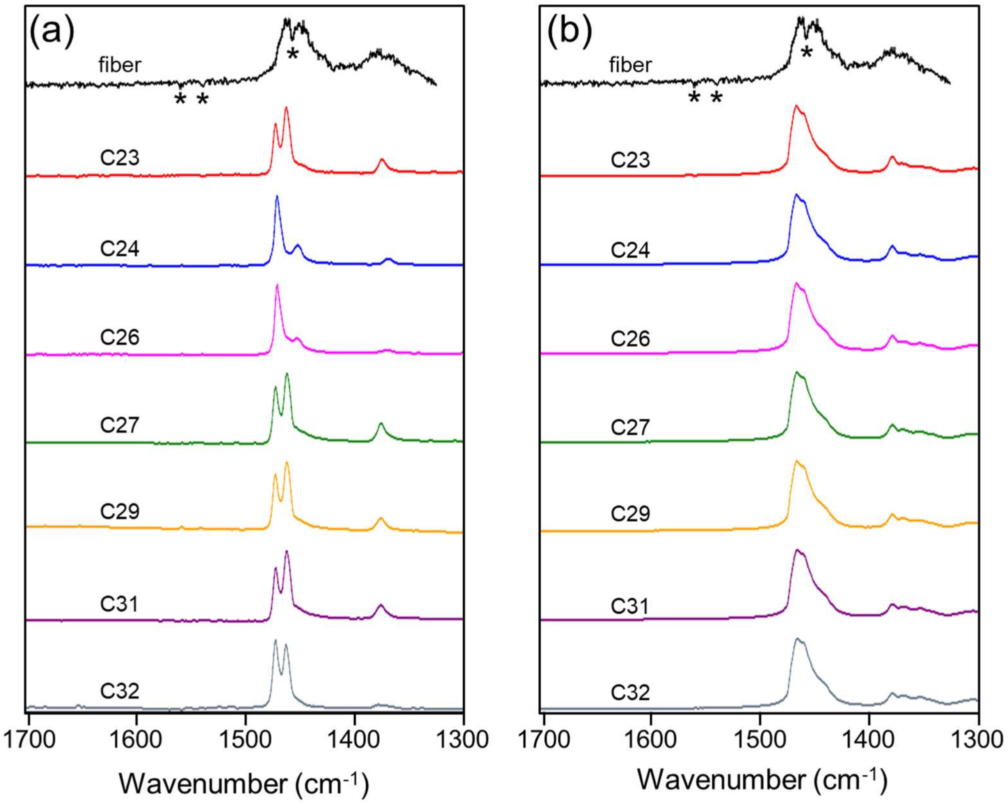

Supplementary Figure Supplementary Figure 11. AFM-IR spectrum of a single fibre compared with several bulk phase spectra of *n*-alkanes from C_{23} to C_{32} recorded in (a) the crystalline solid-state and (b) the amorphous melt phase. (* indicate negative peaks due to atmospheric rotational lines).

It can be seen that the spectrum of the fibre shows broad bands that correspond to methylene bending modes centred at around 1455 cm^{-1} and a broad feature centred around 1375 cm^{-1} attributed to methyl bending vibrations. [28] Albeit the band shape of the methylene bending modes are distorted due to the sharp inverted atmospheric peak, the broad bands observed are consistent with a low crystallinity material as the profile has more similarity to those of the molten *n*-alkanes spectra, and the mixed chain length composition of the fibres will also contribute to the line broadening.

Supplementary Note 8: Supplementary discussion on fibres and hydrocarbon chains

The AFM-IR characterization experiments undoubtedly show that the micrometre long fibres with nanometric width observed in the AFM, SEM and TEM images are composed of hydrocarbon chains. They have a semicrystalline structure, which is fully corroborated by a detailed Raman spectroscopy analysis that shows a close correspondence with the spectra of *n*-alkanes. Therefore, we can envisage that the alkanes that form in the Reaction chamber become agglomerated into a fibre-like structure. Supplementary Figure 2C and Supplementary Figure 3 clearly confirm that the long fibres are already formed when they reach the collection surface.

We show that *n*-alkanes form in the gas-phase in a confined region close to the magnetron inside the Reaction chamber and, as opposed to other magnetron related growth techniques, the growth does not take place on the collecting surface or on the walls activated by surface diffusion of the monomers. However, we do not yet have a clear idea of the intertwisting mechanism that generates the fibres from the alkane chains, although we can speculate that the process may be related to the constricted passage through the nozzle separating the Reaction and Collection chambers (see Supplementary Figure 1). Although we do not know where fibres are formed, Raman spectroscopy performed on the samples collected inside the Reaction chamber (see Supplementary Figures 7 –9) show an almost identical semicrystalline aliphatic nature when compared to those of the Collection chamber, clearly suggesting that the alkanes are formed inside the Reaction chamber.

Another important observation from the Raman spectra of Supplementary Figure 9 is the presence of D and G bands, which are characteristic of amorphous carbon. This also agrees with images such as those shown in Fig. 1c, where both fibres and nanoparticles are seen on the same image and with the XPS spectra. Moreover, in the SEM images some nanoparticles are observed mixed with the fibres, which could support a model such as that presented in Fig. 1, where the carbon nanoparticles formed by the C that has not reacted with hydrogen may act as a catalyst for the formation of the hydrocarbon chains (see Supplementary Figure 12).

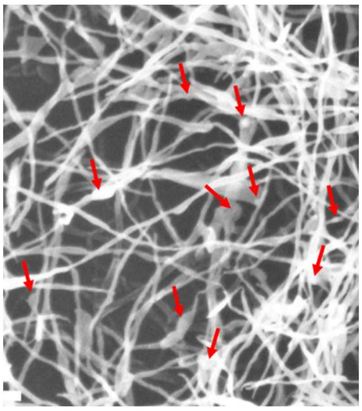

Supplementary Figure 12. SEM image corresponding to a region of the fibre mat. The red arrows indicate C nanoparticles that are mixed with the fibres.

In our model the chain length, is limited by a competition between the polymerization (C_nH_{2n} + CH_2 → $C_{n+1}H_{2n+2}$) and the chain termination (C_nH_{2n} + H → C_nH_{2n+1}) probabilities.

Polymerization requires available molecular hydrogen for the CH_2 radical formation. However, if there is atomic H available the chain will terminate forming a methyl moiety. As the concentration of atomic hydrogen increases, the chain termination probability will increase resulting in shorter chains.

There are two main sources of atomic H: firstly, a proportion of molecular H_2 arrives at the magnetron head and is dissociated. This requires that the flux of molecular hydrogen penetrates into the stream of expanding Ar. We have calculated, very approximately that it could reach between 1-10% of the H_2 concentration introduced. The second source is related to possible chemical reactions producing atomic H from molecular hydrogen (e.g. CH+H_2 → CH_2 + H). Importantly, production of atomic H for any of these mechanisms is proportional to the initial H_2 concentration, and therefore in any case the ratio between H_2 and H would not significantly change.

Therefore, under the current experimental arrangement there is a very narrow range of possibilities to vary this ratio and, as a consequence, we always obtain very similar chain length distributions irrespective of the H_2 flow rate employed, as we have repeatedly observed.

Supplementary Note 9: Ab-initio Density Functional Theory: details & Formation and stability of CH_2.

All ab-initio calculations were carried out by using the plane-wave simulation code CASTEP. [29] One electron wave-functions are expanded by plane-waves with a kinetic energy cut-off of 410 eV. Electronic exchange and correlation (XC) effects were accounted for by the GGA-PBE functional. [30] To model the ion-electron interaction in the H and C atoms, we have used ultra soft pseudopotentials. [31] Tkatchenko-Scheffler semi-empirical dispersion correction scheme [32] has been adopted to account for van der Waals (vdW) interactions in the systems. All the calculations have been performed within a spin-polarized model. The Brillouin zones have been sampled by optimal [2×2×2] Monkhorst-Pack (MP) grids. Atomic positions have been optimized until the maximum net force acting on each atom was < 0.02 eV/Å, and the self-consistent electron density converged up to a precision in the total energy of < 10^{-7} eV.

Minimum reaction paths have been obtained by performing an LST/Optimization calculation followed by a QST maximization.[33] Subsequently, conjugate gradient minimization is performed, and the procedure is repeated until a stationary point is located [climbing-image nudged elastic band method, REF]. The convergence threshold for the norm of the force orthogonal to the reaction path has been set at 0.05 eV/Å. Vibrational calculations on the resulting TSs validate that only one normal mode with negative frequency is obtained.

Formation of alkanes C_nH_{2n+2} proceeds by aggregation of CH_2 units through the following mechanism: The elementary precursors are carbon atoms and molecular hydrogen in a similar amount, and atomic hydrogen dissociated at the plasma as estimated in [2,3]. The experiment takes place in a locally dense Ar atmosphere with an estimated density of $\rho = 10^{18}$ at/cm^3 i.e., an average separation between atoms of $\lambda = 10$ Å. At an estimated temperature of 450 K, the calculated averaged velocities for the different components are $V_{Ar} = 3$ Å /ps, and $V_C = 6$ Å /ps. Therefore, collisions are expected for times below 10 ps (from molecular dynamics simulations, Ar effective scattering cross-section is estimated as $\sigma \approx 4\pi$ Å^2).

From all the possible routes to generate CH_2, the simplest is (T = 450 K),

$C+H_2 \rightarrow CH_2$; $\Delta G = -3.0$ eV (S1)

According to DFT calculations, this is an exothermic reaction with a minimal barrier (below our accuracy level of about 0.01 eV). These results have been confirmed using an extended ($\Delta H = -3.8$ eV) [30] and a localized basis formalism ($\Delta H = -3.5$ eV) [34] and, and agree with other ab-initio and experimental studies reported in the literature. [35–38]

Molecular Dynamics simulations for collisions of C and H_2 show (see Supplementary Movie 1 and next section) the formation of the meta-stable compound CH_2* with a lifetime of $\tau > 10$ ps (estimated from residence times computed from the time spent by the three atoms within a region of about 1.5 Å). Locally, the temperature fluctuates due to the collisions, reaching values of about 10^3 K. The excess energy, approximately 3 eV, is efficiently and quickly redistributed among all the degrees of freedom, which prevents immediate dissociation and increases the lifetime. For the three vibrational degrees of freedom, we compute two A_1 modes with frequencies ≈ 1000 cm-1 and 3100 cm^{-1} and one B_2 mode with ≈ 3300 cm^{-1}. The derived lifetime, which agrees with previous ab-initio calculations, [35] leaves enough time for the complex to interact with a third body (Ar), which helps in stabilizing the configuration by releasing the excess kinetic energy. The long-range attractive van der Waals interaction between Ar and the other

atoms and molecules, and the short-range repulsive one due to the overlap of electronic clouds, are seen to be quite effective in transferring kinetic energy from the short-lived compound to the noble gas atom.

Supplementary Note 10: Molecular Dynamics: details and the reaction $C + H_2 \rightarrow CH_2$.

We investigate the lifetime of the metastable compound CH_2* by *ab-initio* extended Lagrangian Born-Oppenheimer molecular dynamics simulations in the microcanonical ensemble. To produce realistic simulations, which at the same time could span over timescales longer than 10 ps, we utilize a plane-waves basis with on-the-fly generation (OTFG) ultra-soft pseudopotentials, [31] energy cut-off of 325 eV, 10^{-6} eV energy threshold to reach self-consistency, and the same GGA-PBE XC functional and MP wave-functions sampling.[29] Spin-polarized energy bands and a dispersion correction to include long-range van der Waals interactions have been used.[32] Finally, the reaction is simulated inside a 10×10×10 $Å^3$ periodic cell box.

Supplementary Figure 13a shows geometries in time lapses of 1 ps for a head-on collision of H_2 and C (initially well separated by more than 3 Å), moving at thermal velocities corresponding to T=300 K. The evolution of the potential energy and temperature is shown in Supplementary Figure 13b. We observe how the excess energy related to the new bonds' building up is quickly shared between the nine available degrees of freedom in the CH_2* metastable compound. The effect of such equipartition is to increase its lifetime since it takes time to build up an energy fluctuation large enough to take the compound over the barrier of about 3 eV necessary to dissociate it into molecular hydrogen and atomic carbon.

An effective temperature in these simulations takes an average value of about 5800 K (red line in Supplementary Figure 13b). Assuming a frequency attempt of ~3000 cm^{-1} related to the vibrational stretching mode involved in the breaking of the bond, we estimate a lifetime of $\tau \geq \frac{1}{10^{14} e^{-\frac{3}{0.5}}} \approx 10\, ps$, where 3 eV is the barrier height, 0.5 eV corresponds to the thermal energy at 5800K, and 10^{14} s gives the attempt frequency corresponding to 3000 cm^{-1}. Such an estimate for the lifetime is in good agreement with other approaches in the literature.[35] As commented above, our molecular dynamics simulations also support that the compound remains formed during similar time intervals (Supplementary Figure 13a and Supplementary Movie 1).

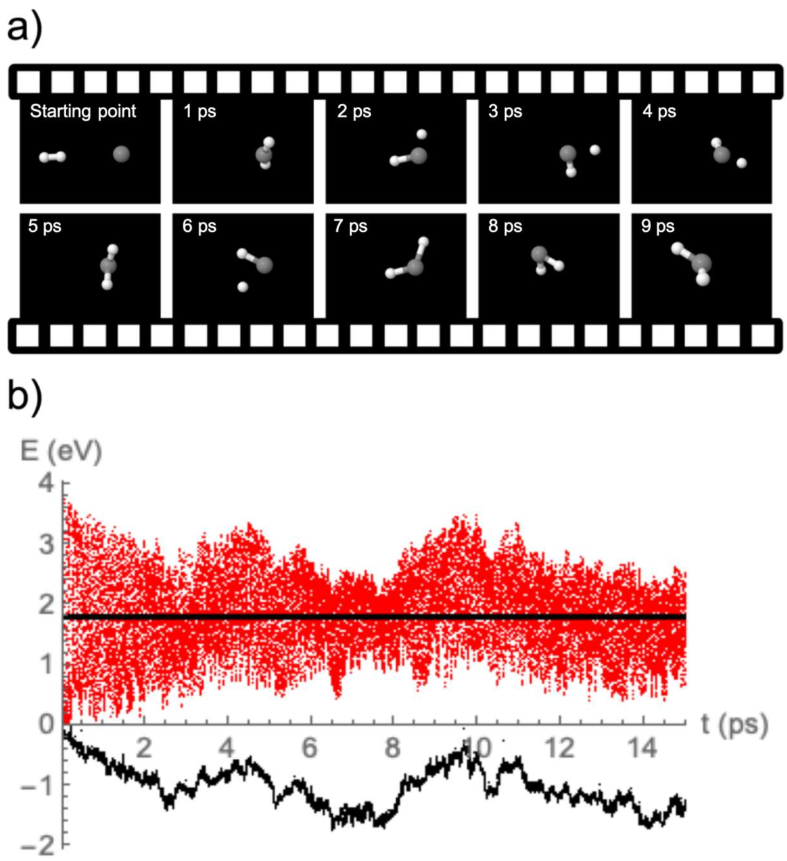

Supplementary Figure Supplementary Figure 13. Molecular Dynamics. C+H_2 → CH_2. a) Evolution of the collision C+H_2 → CH_2 in the first 10 ps shown in frame steps of 1 ps corresponding to the movie (see Supplementary Movie 1). Final panel shows a fully optimized geometrical configuration for CH_2 (T=0 K). b) Evolution of the potential energy (black) and temperature (red), in eV, as a function of time (in ps).

Finally, the role of the noble gas is to remove excess energy, reducing the average temperature in the metastable CH_2*. In the absence of Ar, the maximum T recorded goes up to 14500 K, with an average value of ~6000 K. After interaction with Ar, those values decrease to 9000 and ~3000 K, respectively. The latter value implies an increase in the estimated lifetime by a factor of 20, which further supports our hypothesis that the metastable formation of CH_2 in the Ar atmosphere yields enough opportunities for ulterior growth of long alkane chains.

Supplementary Note 11:- Methylene Polymerization

Despite our arguments above, aggregation of CH_2 monomers in the gas phase to grow an alkane chain would be impeded by the reaction,

$CH_2 + CH_2 \rightarrow C_2H_4$; $\Delta G = -6.3$ eV (S2)

since the formation of a strong C=C double bond creates a barrier of about 3 eV for adding a new CH_2 monomer, $CH_2 + C_2H_4 \rightarrow C_3H_6$. The new C-C bond has a smaller bonding order that does not favour the formation of a long alkane-like structure (without additional constraints, C_3H_6 tends to make a cyclic triangular ring avoiding the formation of a linear-like alkane configuration).

To avoid such a roadblock, we take our inspiration from heterogeneous catalysis. The standard idea is to use the interaction with the catalyst to soften the strong bond which is at the origin of the barrier. Here, the system playing the catalyst's role is merely a H atom, which reduces the barrier to add a new CH_2 unit, stabilizing the linear-like geometry and favouring an alkane formation.

As an example, let us consider the growth of,

$C_2H_5 + CH_2 \rightarrow C_3H_7$; $\Delta G = -3.3$ eV (S3)

The simple addition of a hydrogen atom to C_2H_4, permits the alkane to grow by successive addition of CH_2 groups, with a Gibbs free energy gain of about 3 eV at each step and without a noticeable barrier (Supplementary Figure 14).

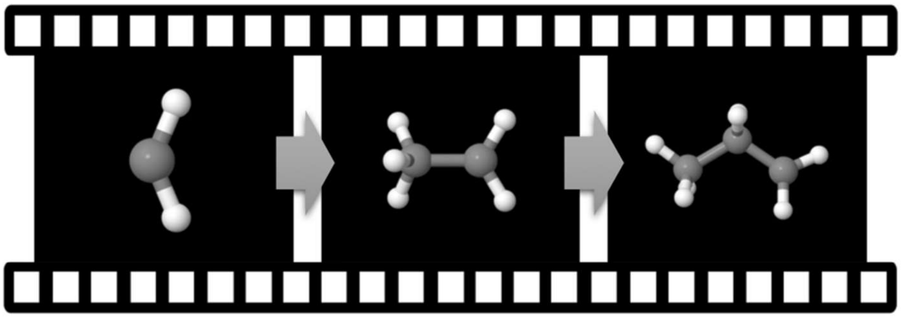

Supplementary Figure 14. Initial steps to polymerize methylene units to form alkanes.

The rationale for the near elimination of the barrier is as follows: CH_2 adopts a geometry characterized by two symmetric C-H bonds of 1.01 Å and an H-C-H angle of 135°. Each C-H bond stores about 3.5 eV. On the other hand, the group CH_2 at the end of C_3H_7 has longer C-H bonds by merely an extra 0.004 Å and, the H-C-H angle adopts the value of 117.8°. A calculation of CH_2 using these parameters reveals an energy difference of +0.114 eV that needs to be accommodated to join a gas-phase CH_2 molecule to C_2H_5 and form C_3H_7. Such destabilization of CH_2 to join the longer chain would be in the origin of a possible barrier. Compared with the energy gain of ≈ 3 eV due to the newly formed C-C bond, such a slight worsening of the internal energy in CH2 can be neglected. It is easily accommodated in our calculations by incremental geometry modifications, resulting in a negligible zero-barrier process.

This argument repeats itself for successive additions of CH_2 fragments to build up a long alkane chain. It becomes physically similar but even more robust when the initial C-C weakening is performed by attaching the molecule to another C atom; e.g. as suggested in Supplementary Figure 15, where an amorphous C nanoparticle has been used to anchor the seed for growing the alkane. The alkane ceases growing when a H atom radical is attached to the alkane chain's free chain.

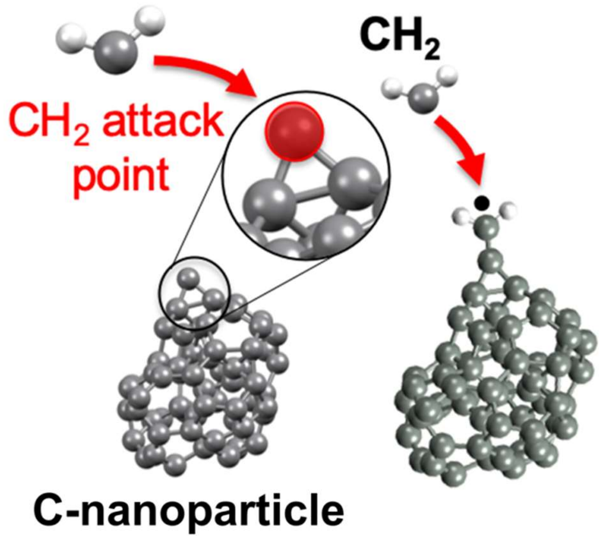

Supplementary Figure 15. Nanoparticle anchoring CH_2. The C-CH_2 bond allows the continued growing of the alkane.

Supplementary Note 12: Carbon nanoparticle considerations and modelling

Carbon nanoparticles are a key point in our model. They are formed in the gas phase and we have shown with previous experiments [3] that they consist of amorphous material, as seen by TEM measurements. The non-crystalline nature of the nanoparticles was proposed because they are formed by aggregation and therefore, a metastable amorphous structure can be formed. [39]

To model the particles, we have constructed a set of disordered starting-point configurations, which subsequently have been fully-relaxed by DFT to reach zero-forces equilibrium. The modelling protocol can be summarized as follows: i) Aggregates with a different number of randomly distributed C atoms have been constructed under the condition of having first-neighbour C-C distances between 1.2 and 1.5 Å (a distance range that comprises typical single, double and triple C-C bond-lengths) ; ii) A “structural shaking” in the C—C distances of about 15% was applied to reinforce the nanoparticles’ final amorphous character, which was subsequently fully DFT-relaxed (details explained in Supplementary Note 9); iii) Finally, we initiated a full relaxation process where an additional 5% “shaking” was applied to end up with equilibrium structures. This strategy has been applied to different C_n cluster sizes (n=20, 60 and 100). For C_{60}, all the final configurations had total energies above 8 eV compared to the highly-symmetric isomer I_h Buckminster-fullerene, whose formation is thermo-kinetically hindered at the low temperatures present in our atomic aggregation experiments. We note that the average first-neighbour C—C distance in all the amorphous nanoparticles range between 1.31 and 1.35 Å, which indicates that a majority of bonds have Pauling bond orders above 2 and display low remaining reactivity. However, we find some highly reactive unsaturated C atoms on the outer shell, especially if compared with the most stable C_{60} and C_{100} cage-like aggregates. We have concentrated on C_{60} amorphous nanoparticles because they are already large enough to accommodate and dissipate the energy released while the alkane grows. At the same time, its size makes a computationally affordable model for our time-consuming theoretical protocol. Nonetheless, we have checked that the shape and size of the amorphous nanoparticle structures selected to perform the alkane growth do not significantly affect the thermokinetic reactions.

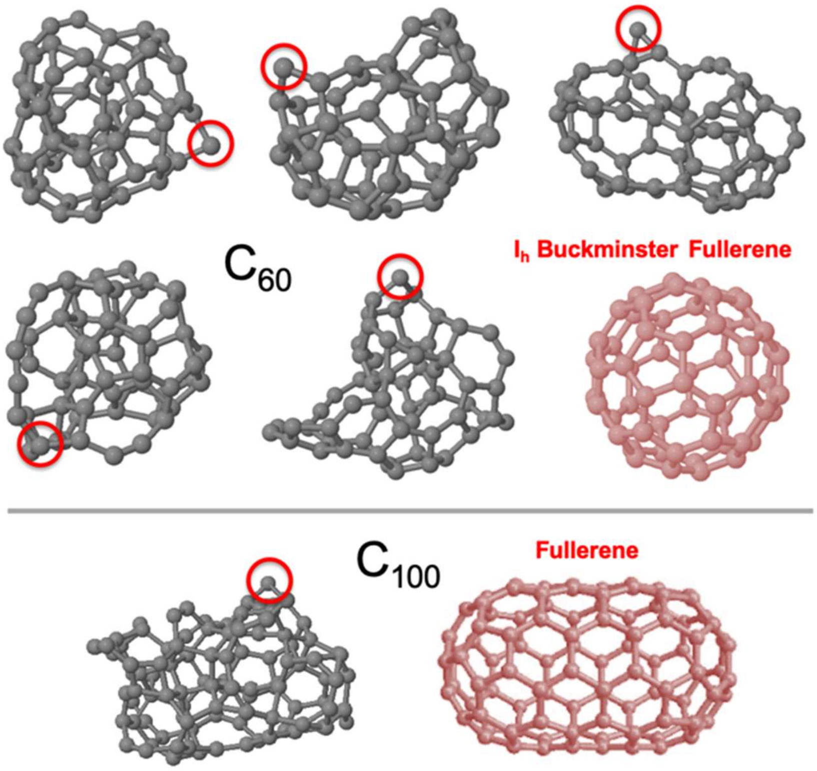

Supplementary Figure 16. Pictorial sketch of the DFT computed structures of some amorphous nanoparticles obtained by our modelling protocol as compared with their high-symmetry counterparts. Red circles mark external high-reactivity points.

Supplementary Note 13: Alkane Detachment Mechanism

The detachment process of the as-grown alkane (A) species from the nanoparticle (NP) may be favoured, induced and driven by several synergistic routes, among which we could mention collisions with Ar, the entropic instability inherent to large molecules leading to its breaking into smaller pieces, or a stretching phonon activated by the environmental thermal bath inducing the structural detachment of the alkane.

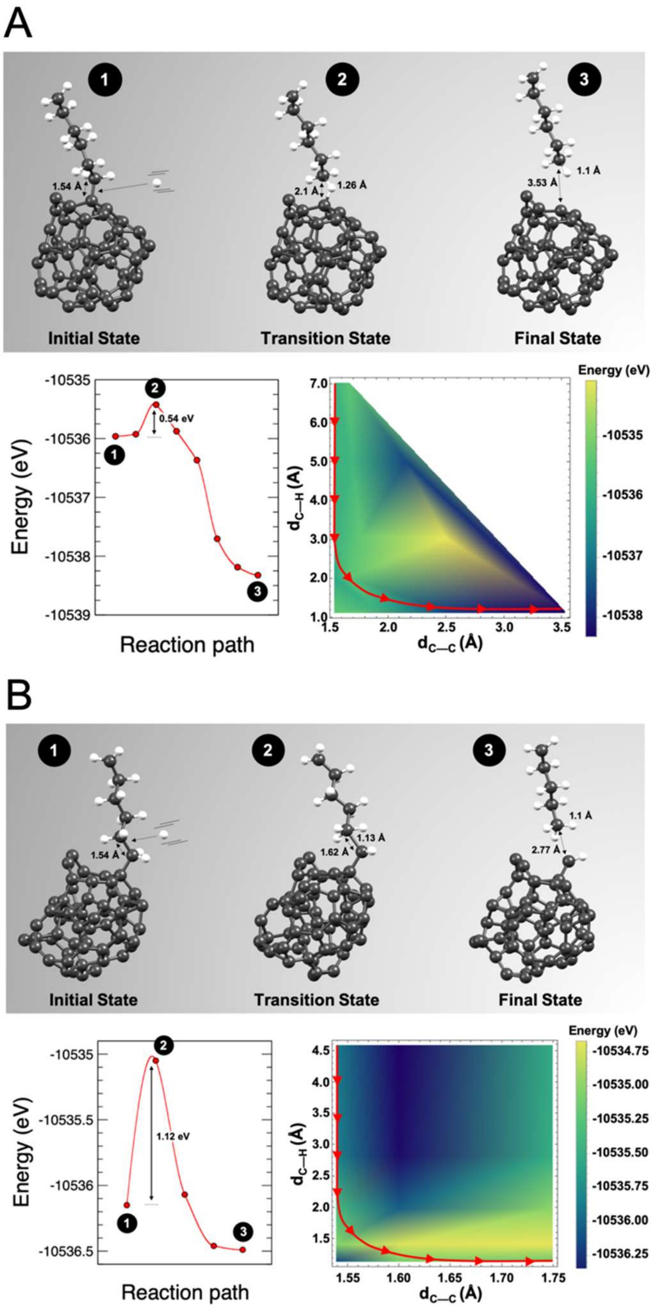

Supplementary Figure 17. Proposed mechanisms to detach an alkane grown on a model amorphous C_{60} nanoparticle by interaction of an "attacking" H atom to weaken a C–C bond. Two models have been proposed for which the initial state, the transition state and the final state, and their most representative distances are given: A) an atomic H attacks the linking C–C bond connecting the alkane to the nanoparticle, and B) an atomic H attacks the first internal C–C bond

within the alkane closer to the NP. Most favourable reaction path vs. total energy, and colour-mapped 2D potential surface are shown in both cases, indicating superimposed to the colour maps the reaction path. Thermokinetic barriers result in 0.54 and 1.12 eV for the A and B cases, respectively, and net reaction enthalpy gains are -2.29 and -0.35 eV.

We quantify the probability of detachment by referring to a previous work on etching C–C bonds by atomic H, [40] i.e. we use DFT to compute the weakening of a C–C single bond by interaction with an "attacking" H atom. Two scenarios have been considered here: i) the single C–C bond linking the as-grown alkane to the NP, and ii) some C–C bond within the alkane (we assume that all store a similar amount of energy, around 3.8 eV). For the systems of interest, we find that the substitution of a C–C bond by a C–H one results in a thermodynamic gain in the range -0.5 to -2 eV. Therefore, our main concern is to estimate the energy barrier generated upon the concerted destruction of the C–C bond while creating the new C–H one. We find for the C–C link between A–NP a thermo-kinetic barrier of 0.54 eV and, for the first internal C–C in A, a barrier of 1.12 eV (see details below and Supplementary Figure 17). We estimate the probability of picking up a thermal fluctuation large enough to overcome the barrier from a Boltzmann distribution, and we estimate the frequency attempt from some typical C–H stretching phonon (around 3000 cm^{-1}). At an environmental temperature of 600 K, this results in 382 ps and 28 ms for the barriers above, which shows that the alkane detachment process has a large probability of happening in the typical residence time of the NP of around several seconds.

For both cases, the computed minimum reaction path associated to these mechanisms shows that, as the "attacking" H approaches the C–C bond, first it has to overcome the electrostatic barrier of surrounding H atoms forming the alkane. Simultaneously, in a synergistic effect which helps to minimize the barrier, such H atom starts forming C–H bonds, which favours stretching of the C–C single bond-length, from its equilibrium distance of 1.54 Å up to a distance for which the alkane is wholly detached from the nanoparticle (see Supplementary Figure 17). The final most stable product is the NP plus a detached A that has integrated into its structure the attacking H atom, leaving a saturated terminal –CH_3 group in the alkane.

Supplementary Note 14: Reconstruction of the flow geometry in the magnetron head

We have performed a direct numerical simulation of the Ar dynamics using the magnetron geometry boundary conditions to uncover possible regions able to capture the sputtered C atoms near the target surface and then release the products. The experimental geometry leads us to estimate a Reynolds number, Re ~20-30 and a Mach number, Ma < 0.007. At these values, unsteadiness and compressibility in the flow are not expected to play a significant role, which prompts us to use a steady, incompressible simulation of the gas phase flow that has allowed us to uncover a previously unknown trap-and-release mechanism in the flow. Given the size of C atoms, inertial effects in their motion are also negligible. These conditions give rise to a temporary trapping mechanism that is already present in the steady, 3D streamline geometry of fluid particles. Next, we focus on describing the mechanism for the Re = 20 case but a similar analysis (see next section) for the Re = 30 case shows that an analogous trap-and-release mechanism persists, even with a significantly altered streamline geometry.

Despite the perfect cylindrical symmetry of the geometry, the incompressible simulation yields a converged solution with a four-fold broken symmetry. Nevertheless, sectional streamlines, obtained from projecting velocities onto cross-sections of the magnetron area, suggest a perfectly closed, toroidal recirculation zone without any possibility for capturing and releasing C atoms (Fig. 4A, main text). Other applicable flow-visualization tools, such as finite-time Lyapunov exponents [41,42] or Poincaré maps offer no further insights. However, a detailed tracking of judiciously chosen streamlines reveals three topological features shown in the calculations of Fig. 4A-B and schematically drawn in Fig. 4C and Supplementary Figure 18) The main stream (red line), which is invariant and represents the main Ar jet, b) a dome-shaped surface (orange line) resembling a deformed umbrella, serving as the 2D stable manifold of a saddle-type stagnation point [43] near the vertical symmetry line (at 6.3 mm from the target surface), and c) four nearly planar, invariant vertical surfaces (subsequently referred to as planes), oriented in the radial directions and separated from each other by an angle of about 45 degrees. Of these surfaces, Type-1 is the 2D stable manifold of a saddle-focus-type point. Near this plane, the dome-shaped surface runs into the orifice in backward time, preventing fluid particles under the dome from escaping into the main jet. Instead, these particles (green line) are attracted to the Type-1 plane near the orifice, which forces them to spiral towards the saddle-focus fixed point. The fluid particles are then ejected by the one-dimensional unstable manifold of the fixed point towards an adjacent, Type 2-plane. The ejected particles approach another saddle-focus fixed point in the Type-2 plane along its one-dimensional stable manifold and then subsequently spiral out along the Type-2 plane, which is the 2D unstable manifold of that fixed point. Near this plane, the dome-shaped surface does not reach into the orifice in backward time and hence does not form a barrier to the outward-spiralling particles. Rather, these particles accumulate onto a 2D separation surface emanating from the circular orifice, pass by the top of the dome along this separation surface, enter the main stream and subsequently leave the target. The whole process can be visualized in the Supplementary Movie 2.

This interplay between Type-1 and Type-2 planes results in the temporary entrapment of a large number of particles. In fact, arbitrarily long trapping times can arise for particles starting arbitrarily close to Type-1 planes. For particles released uniformly around the sputtering zone,

we compute the distribution of their residence times (see Fig 4D). The expected value of the carbon atom's trapping time is about 0.83 seconds, which enables reactions that otherwise could not take place for trajectories through the magnetron without trapping. To the best of our knowledge, this trapping-release mechanism in steady flows has not been previously documented in fluid dynamics literature, and hence is of interest in its own right.

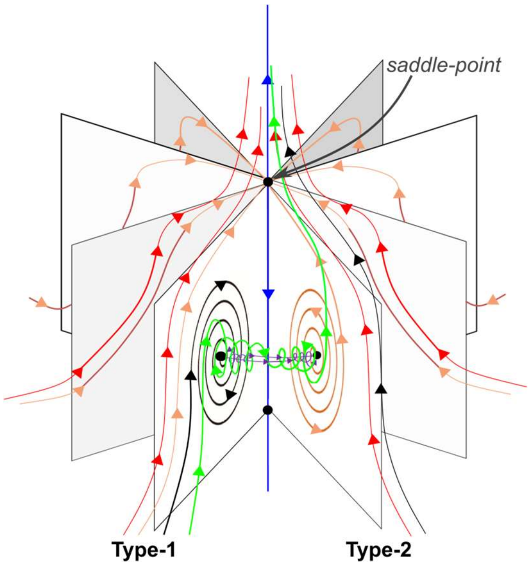

Supplementary Figure 18. Schematic of special streamlines and invariant planes forming the trap-and-release mechanism. Green line: C trajectory being trapped and released. Purple lines: broken heteroclinic connection between stable and unstable spirals. Red lines: Intersection of the 2D stable manifold of the saddle-type fixed point with the planes. Black line: Intersection of the 2D separation surface with the plane. Orange line: Intersection of 2D separation surface emanating from the circular bottom with the plane.

In Supplementary Figure 19, we show in white the two-dimensional stable manifold of the saddle-type stagnation point [43] which lies approximately on the axis of symmetry of the computational domain. To obtain the position of this stagnation point with the highest possible accuracy given the current resolution, we track down the computational cell which has inward-pointing velocities on each of its faces. The centre of this cell is considered the approximate position of the stagnation point and after using a small sphere of initial conditions around it, we construct the stable manifold by backward-time advection.

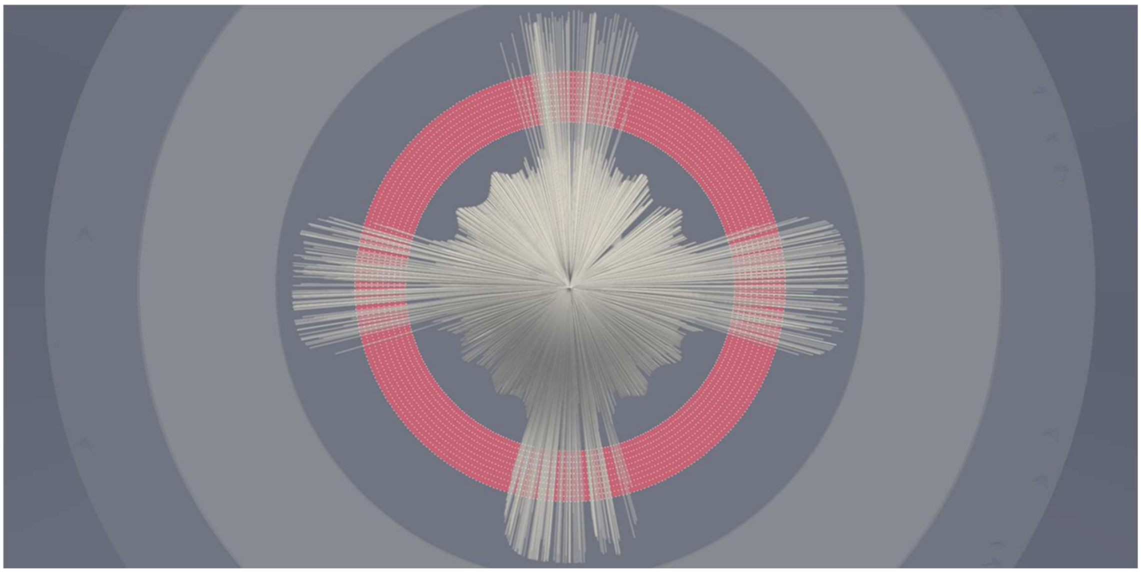

Supplementary Figure 19. Released positions of the carbon atoms superimposed with the 2D stable manifold of the saddle-type stagnation point (Re=20).

The red ring with an inner diameter of 26 mm and an outer diameter of 34 mm is located close to the bottom wall, closely approximating the sputtering zone of the carbon atoms. We estimate the residence-time distribution of these atoms in the magnetron using 360 x 20 initial conditions placed uniformly along the azimuthal and radial directions, respectively.

Supplementary Figure 20 shows sectional streamlines (a frequently used heuristic indicator of 3D flow geometry) on the two Type-1 planes of Fig. 4B (main text). The picture remains the same in all other radial planes, suggesting a toroidal vortex that covers the entire recirculation zone. Because of the steadiness of the flow, however, atoms that start inside this region will remain there trapped forever. This is in stark contrast with what is actually happening when we consider the full three-dimensional field.

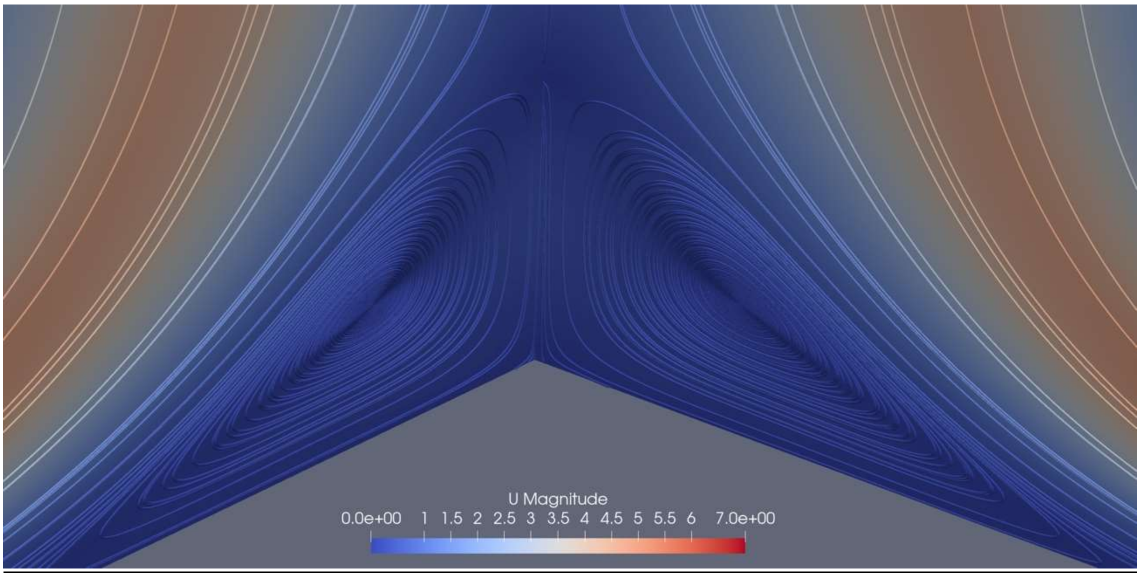

Supplementary Figure 20. Sectional streamlines on two Type-1 planes (Re = 20).

Similarly, techniques such as the finite-time Lyapunov exponents (FTLE) [41,42] fail to reveal any trapping mechanism allowing enough residence time for the chemical reactions to take place. Specifically, for the computation of the FTLE, we used a grid of 250 x 250 initial conditions advected backward in time from a plane at y = 2.3 mm. This is slightly below the midplane between the bottom wall and the plane containing the saddle-type stagnation point. The integration continues approximately until the trajectories reach the orifice (Supplementary Figure 21). shows the result of this computation without suggesting any prominent feature other than the separation profile emanating from the edge of the circular orifice.

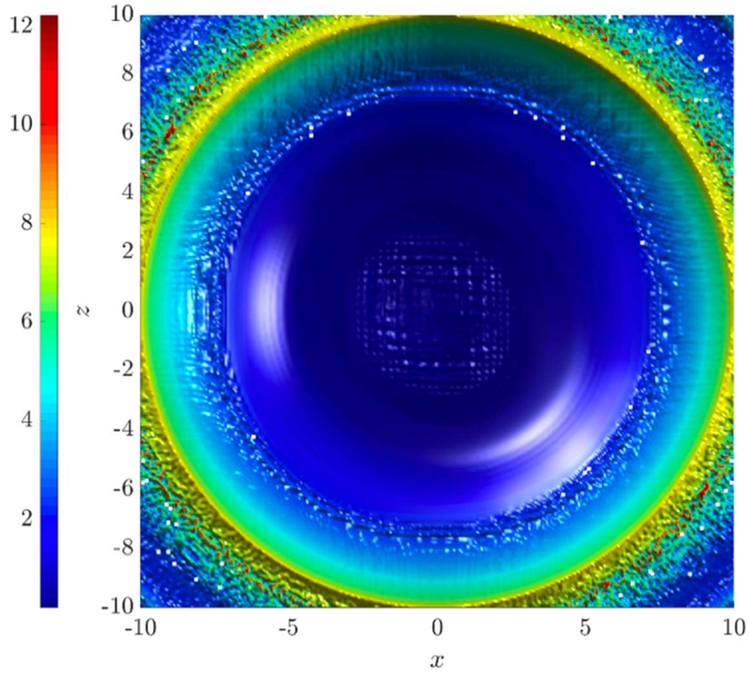

Supplementary Figure 21. Finite-time Lyapunov exponent field on a plane located at y=2.3 mm above the magnetron surface covering the recirculation zone.

Likewise, Supplementary Figure 22 depicts the Poincaré maps (or first-return maps) [43] produced using a grid of 200 x 150 initial conditions uniformly placed along the two Type-1 planes of Fig. 4B Again, no further insight is gained using this approach because the flow does not generate enough trajectories that revisit the same plane.

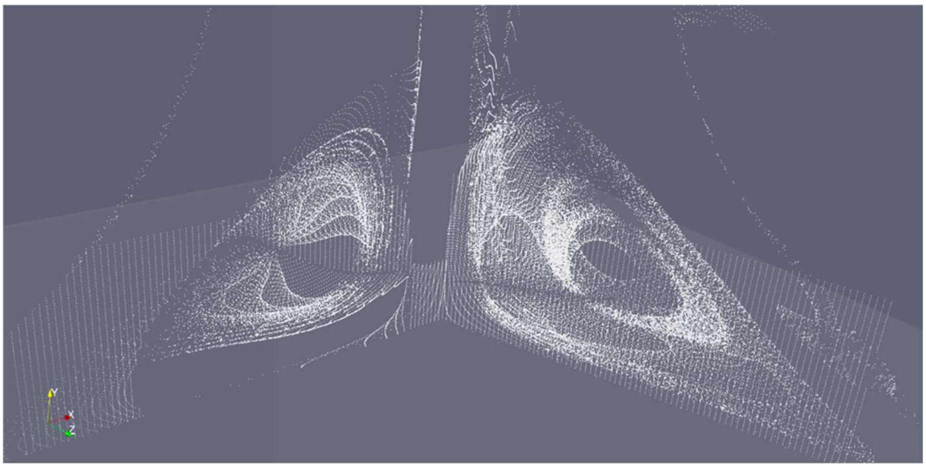

Supplementary Figure 22. Poincaré maps on the two Type-1 planes of Fig. 1.

As pointed out in the main text, where the trapping-release mechanism for the Re = 20 case is described, we have also simulated the Re = 30 case. In contrast to the Re = 20 flow, the converged solution here reveals a flow with a two-fold broken symmetry, the topological skeleton of which is depicted in Supplementary Figure 23. This steady flow consists of five fixed points all of which are of saddle type. For each of the fp2 fixed points representative fluid particles emanating from their neighbourhood spiral around their 1D unstable manifold connecting the fp2 fixed points with the fp1 and fp3 fixed points. The fp1 fixed point has a 2D unstable manifold which is nearly planar and along which the approaching particles spiral out of the magnetron resembling the Re = 20 case. In contrast to that, the two-dimensional unstable manifolds of the fp3 fixed points perturb to the green surfaces of Supplementary Figure 24 which attract fluid particles stemming from the neighbourhood of the fp2 fixed points before they start spiralling out to reach the main Ar flow.

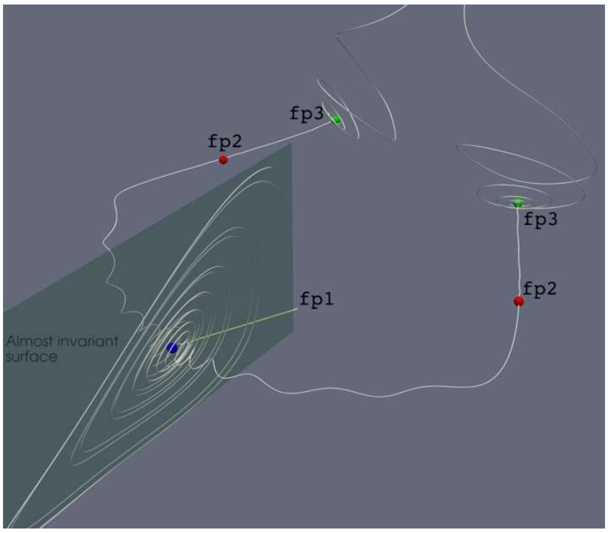

Supplementary Figure 23. Skeleton of the Re = 30 case depicting the symmetry-breaking about the nearly planar two-dimensional unstable manifold of the fp1 fixed point. Trajectories in the neighbourhood of the fp2 fixed points show the different idealized routes carbon atoms might follow to escape the trapping regions.

The building block of the trap-and-release mechanism in this case comprises the stable manifolds of the fp2 fixed points shown in pale white on Supplementary Figure 24. The extent of these two-dimensional surfaces varies significantly with some branches nearing the orifice and others remaining very close to the centre of the magnetron. Together with the almost planar, two-dimensional unstable manifold of the fp1 fixed point, these surfaces entrap fluid particles that originate inside them for arbitrarily long times before they are eventually released through the routes depicted in Supplementary Figure 24. Using the same grid of initial conditions as in the previous case, we obtain a highly asymmetrical profile of residence times (Supplementary Figure 25), which agrees with the main flow features. According to this, the expected value of the carbon atom's trapping time is about 0.7 seconds providing enough time for them to react before exiting the magnetron vicinity.

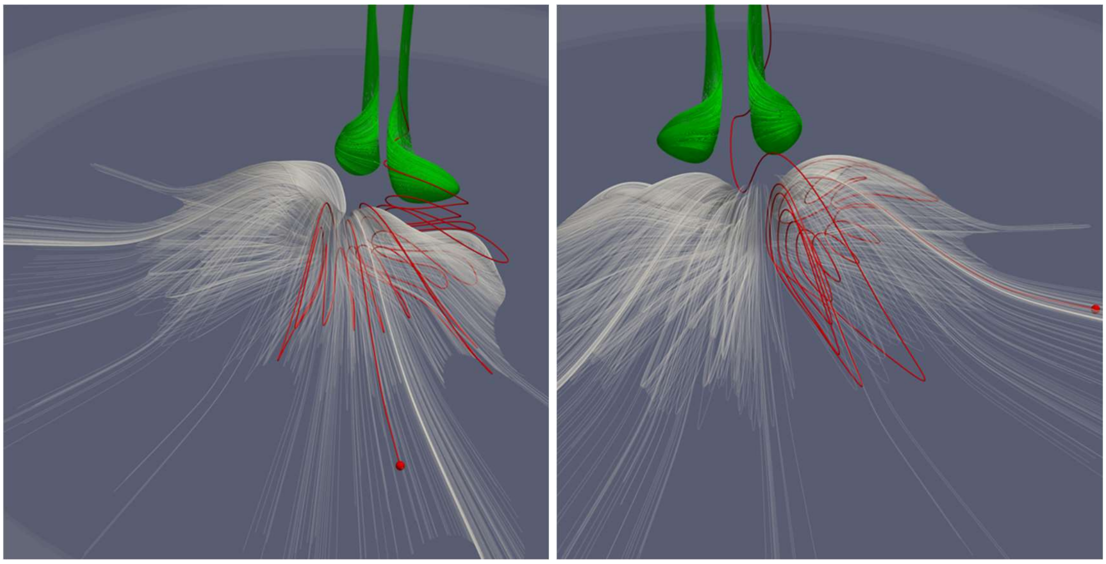

Supplementary Figure 24. Two-dimensional stable manifolds of the fp2 fixed points (pale white surfaces) along with the two-dimensional unstable manifolds of the fp3 fixed points (green surfaces). Trap-and-release events for two different C atoms released in the flow from the injection ring of Supplementary Figure 16 are shown by the red streamlines (Re=30).

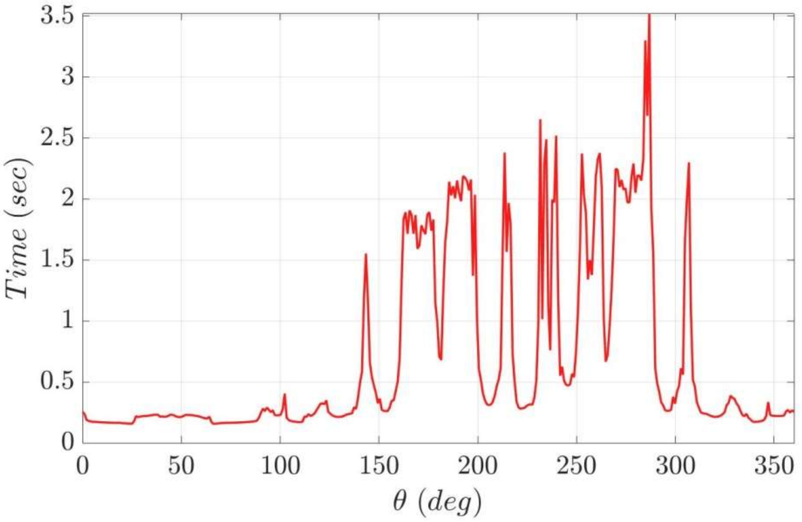

Supplementary Figure 25. Residence time distribution as a function of azimuth along the injection ring shown on Supplementary Figure 16 (Re=30).

SUPPLEMENTARY REFERENCES

1. Martínez, L. *et al.* Precisely controlled fabrication, manipulation and in-situ analysis of Cu based nanoparticles. *Sci. Rep.* **8**, 7250 (2018).
2. Martínez, L. *et al.* Prevalence of non-aromatic carbonaceous molecules in the inner regions of circumstellar envelopes. *Nat. Astron.* **4**, 97–105 (2020).
3. Santoro, G. *et al.* The Chemistry of Cosmic Dust Analogs from C, C2, and C2H2 in C-rich Circumstellar Envelopes. *Astrophys. J.* **895**, 97 (2020).
4. de la Peña O'Shea, V. A., Campos-Martín, J. M. & Fierro, J. L. G. Strong enhancement of the Fischer–Tropsch synthesis on a Co/SiO2 catalyst activate in syngas mixture. *Catal. Commun.* **5**, 635–638 (2004).
5. de la Peña O'Shea, V. A., Álvarez-Galván, M. C., Campos-Martín, J. M. & Fierro, J. L. G. Fischer–Tropsch synthesis on mono- and bimetallic Co and Fe catalysts in fixed-bed and slurry reactors. *Appl. Catal. A Gen.* **326**, 65–73 (2007).
6. Kashtanov, P. V, Smirnov, B. M. & Hippler, R. Magnetron plasma and nanotechnology. *Physics-Uspekhi* **50**, 455 (2007).
7. Rai, A. *et al.* Operational limit of a planar DC magnetron cluster source due to target erosion. *Nucl. Instruments Methods Phys. Res. Sect. B Beam Interact. with Mater. Atoms* **316**, 6–12 (2013).
8. Bettini, L. G., Galluzzi, M., Podestà, A., Milani, P. & Piseri, P. Planar thin film supercapacitor based on cluster-assembled nanostructured carbon and ionic liquid electrolyte. *Carbon N. Y.* **59**, 212–220 (2013).
9. Accolla, M. *et al.* Silicon and hydrogen chemistry under laboratory conditions mimicking the atmosphere of evolved stars. *arXiv Prepr. arXiv2011.01581* (2020).
10. aiichi SHIKAMA, Shinichiro KADO, Yousuke KUWAHARA, Kiminori KURIHARA, F. S. and S. T. Fulcher-α Band Spectra in Mixed Hydrogen Isotope Plasmas. *Plasma Fusion Res* **2**, S1045 (2007).
11. Dry, M. E. High quality diesel via the Fischer–Tropsch process – a review. *J. Chem. Technol. Biotechnol.* **77**, 43–50 (2002).
12. Saeidi, S., Talebi Amiri, M., Saidina Amin, N. A. & Rahimpour, M. R. Progress in Reactors for High-Temperature Fischer–Tropsch Process: Determination Place of Intensifier Reactor Perspective. *Int. J. Chem. React. Eng.* **12**, 639–664.
13. Moulder, J. F. Handbook of X-ray photoelectron spectroscopy. (1995).
14. Snyder, R. G. Vibrational spectra of crystalline n-paraffins: Part I. Methylene rocking and wagging modes. *J. Mol. Spectrosc.* **4**, 411–434 (1960).
15. Gall, M. J., Hendra, P. J., Peacock, O. J., Cudby, M. E. A. & Willis, H. A. The laser-Raman spectrum of polyethylene: The assignment of the spectrum to fundamental modes of vibration. *Spectrochim. Acta Part A Mol. Spectrosc.* **28**, 1485–1496 (1972).
16. Hendra, P. J., Jobic, H. P., Marsden, E. P. & Bloor, D. The vibrational spectrum of polyethylene—III. Polarized Raman spectra of "single crystal texture" polyethylene and a single crystal of C23H48. *Spectrochim. Acta Part A Mol. Spectrosc.* **33**, 445–452 (1977).
17. Abbate, S., Zerbi, G. & Wunder, S. L. Fermi resonances and vibrational spectra of crystalline and amorphous polyethylene chains. *J. Phys. Chem.* **86**, 3140–3149 (1982).
18. Sagitova, E. A. *et al.* Regularity modes in Raman spectra of polyolefins: Part II. Polyethylene and ethylene copolymers. *Vib. Spectrosc.* **84**, 139–145 (2016).
19. Tomba, J. P., Silva, L. I., García Genga, M., Barrera Galland, G. & Perez, C. J. Characterizing chemical composition of polyolefin-based copolymers from spectral features in the C—H stretching region. *J. Raman Spectrosc.* **50**, 576–586 (2019).
20. Strobl, G. R. & Hagedorn, W. Raman spectroscopic method for determining the

crystallinity of polyethylene. *J. Polym. Sci. Polym. Phys. Ed.* **16**, 1181–1193 (1978).
21. Ferrari, A. C. & Robertson, J. Interpretation of Raman spectra of disordered and amorphous carbon. *Phys. Rev. B* **61**, 14095–14107 (2000).
22. Dazzi, A., Prazeres, R., Glotin, F. & Ortega, J. M. Local infrared microspectroscopy with subwavelength spatial resolution with an atomic force microscope tip used as a photothermal sensor. *Opt. Lett.* **30**, 2388–2390 (2005).
23. Lu, F. & Belkin, M. A. Infrared absorption nano-spectroscopy using sample photoexpansion induced by tunable quantum cascade lasers. *Opt. Express* **19**, 19942–19947 (2011).
24. Centrone, A. Infrared Imaging and Spectroscopy Beyond the Diffraction Limit. *Annu. Rev. Anal. Chem.* **8**, 101–126 (2015).
25. Dazzi, A. & Prater, C. B. AFM-IR: Technology and Applications in Nanoscale Infrared Spectroscopy and Chemical Imaging. *Chem. Rev.* **117**, 5146–5173 (2017).
26. Lu, F., Jin, M. & Belkin, M. A. Tip-enhanced infrared nanospectroscopy via molecular expansion force detection. *Nat. Photonics* **8**, 307–312 (2014).
27. Mathurin, J. *et al.* How to unravel the chemical structure and component localization of individual drug-loaded polymeric nanoparticles by using tapping AFM-IR. *Analyst* **143**, 5940–5949 (2018).
28. Colthup, N. B., Daly, L. H. & Wiberley, S. E. CHAPTER 5 - METHYL AND METHYLENE GROUPS. in (eds. Colthup, N. B., Daly, L. H. & Wiberley, S. E. B. T.-I. to I. and R. S. (Third E.) 215–233 (Academic Press, 1990). doi:https://doi.org/10.1016/B978-0-08-091740-5.50008-9.
29. Clark, S. J. *et al.* First principles methods using CASTEP. *Zeitschrift für Krist. - Cryst. Mater.* **220**, 567–570.
30. Perdew, J. P., Burke, K. & Ernzerhof, M. Generalized Gradient Approximation Made Simple. *Phys. Rev. Lett.* **77**, 3865–3868 (1996).
31. Vanderbilt, D. Soft self-consistent pseudopotentials in a generalized eigenvalue formalism. *Phys. Rev. B* **41**, 7892–7895 (1990).
32. Tkatchenko, A. & Scheffler, M. Accurate Molecular Van Der Waals Interactions from Ground-State Electron Density and Free-Atom Reference Data. *Phys. Rev. Lett.* **102**, 73005 (2009).
33. Govind, N., Petersen, M., Fitzgerald, G., King-Smith, D. & Andzelm, J. A generalized synchronous transit method for transition state location. *Comput. Mater. Sci.* **28**, 250–258 (2003).
34. Frisch, M. *et al.* gaussian 09, Revision d. 01, Gaussian. *Inc., Wallingford CT* **201**, (2009).
35. Harding, L. B., Guadagnini, R. & Schatz, G. C. Theoretical studies of the reactions hydrogen atom + methylidyne -> carbon + hydrogen and carbon + hydrogen-> methylene using an ab initio global ground-state potential surface for methylene. *J. Phys. Chem.* **97**, 5472–5481 (1993).
36. Krasnokutski, S. A. *et al.* ULTRA-LOW-TEMPAB - The reactions of carbon atoms with dihydrogen have been investigated in liquid helium droplets at T = 0.37 K. A calorimetric technique was applied to monitor the energy released in the reaction. The barrierless reaction between a singl. *Astrophys. J.* **818**, L31 (2016).
37. Henning, T. K. & Krasnokutski, S. A. Experimental characterization of the energetics of low-temperature surface reactions. *Nat. Astron.* **3**, 568–573 (2019).
38. Zhang, L., Liu, D., Yue, D., Song, Y. & Meng, Q. Dynamics of H(2 S) + CH(X 2Π) reactions based on a new CH2(${\tilde{X}}^{3}A^{\prime\prime} $) surface via extrapolation to the complete basis set limit. *J. Phys. B At. Mol. Opt. Phys.* **53**, 95202 (2020).
39. Sinitsa, A. S., Lebedeva, I. V, Popov, A. M. & Knizhnik, A. A. Transformation of Amorphous Carbon Clusters to Fullerenes. *J. Phys. Chem. C* **121**, 13396–13404 (2017).
40. Martínez, J. I., Martín-Gago, J. A., Cernicharo, J. & de Andres, P. L. Etching of Graphene in a Hydrogen-rich Atmosphere toward the Formation of Hydrocarbons in Circumstellar

Clouds. *J. Phys. Chem. C* **118**, 26882–26886 (2014).
41. Haller, G. Lagrangian Coherent Structures. *Annu. Rev. Fluid Mech.* **47**, 137–162 (2015).
42. Haller, G. Distinguished material surfaces and coherent structures in three-dimensional fluid flows. *Phys. D Nonlinear Phenom.* **149**, 248–277 (2001).
43. Guckenheimer, J. & Holmes, P. *Nonlinear Oscillations, Dynamical Systems, and Bifurcations of Vector Fields*. (Springer, New York, NY). doi:https://doi.org/10.1007/978-1-4612-1140-2.
