# Supplementary material for: Metal-catalyst-free gas-phase synthesis of long-chain hydrocarbons
Source: Nat Commun. 2021 Oct 12;12:5937. doi: 10.1038/s41467-021-26184-0 (PMC8511129; doi:10.1038/s41467-021-26184-0)
Supplement: Supplementary file 3 — Description of Additional Supplementary Files [file 41467_2021_26184_MOESM3_ESM.pdf]

Description of Additional Supplementary Files

File Name: Supplementary Movie 1
Description: Molecular Dynamics simulations of the first 10ps of the interaction of C + H_2 to form the metastable CH_2 radical

File Name: Supplementary Movie 2
Description: Schematic representation of the calculated trajectories of an atom ejected from the magnetron.
